# Future habitat suitability for coral reef ecosystems under global warming and ocean acidification

**DOI:** 10.1111/gcb.12335

**Published:** 2013-10-08

**Authors:** Elena Couce, Andy Ridgwell, Erica J Hendy

**Affiliations:** * School of Geographical Sciences, University of BristolBristol, BS8 1SS, UK; † School of Earth Sciences, University of BristolBristol, BS8 1RJ, UK; ‡ School of Biological Sciences, University of BristolBristol, BS8 1UG, UK

**Keywords:** Bioclimatic Envelope Modeling, boosted regression trees, coral reef ecosystems, global warming, MaxEnt, maximum entropy, ocean acidification

## Abstract

Rising atmospheric CO_2_ concentrations are placing spatially divergent stresses on the world's tropical coral reefs through increasing ocean surface temperatures and ocean acidification. We show how these two stressors combine to alter the global habitat suitability for shallow coral reef ecosystems, using statistical Bioclimatic Envelope Models rather than basing projections on any *a priori* assumptions of physiological tolerances or fixed thresholds. We apply two different modeling approaches (Maximum Entropy and Boosted Regression Trees) with two levels of complexity (one a simplified and reduced environmental variable version of the other). Our models project a marked temperature-driven decline in habitat suitability for many of the most significant and bio-diverse tropical coral regions, particularly in the central Indo-Pacific. This is accompanied by a temperature-driven poleward range expansion of favorable conditions accelerating up to 40–70 km per decade by 2070. We find that ocean acidification is less influential for determining future habitat suitability than warming, and its deleterious effects are centered evenly in both hemispheres between 5° and 20° latitude. Contrary to expectations, the combined impact of ocean surface temperature rise and acidification leads to little, if any, degradation in future habitat suitability across much of the Atlantic and areas currently considered ‘marginal’ for tropical corals, such as the eastern Equatorial Pacific. These results are consistent with fossil evidence of range expansions during past warm periods. In addition, the simplified models are particularly sensitive to short-term temperature variations and their projections correlate well with reported locations of bleaching events. Our approach offers new insights into the relative impact of two global environmental pressures associated with rising atmospheric CO_2_ on potential future habitats, but greater understanding of past and current controls on coral reef ecosystems is essential to their conservation and management under a changing climate.

## Introduction

Anthropogenic climate change has emerged as a serious global-scale threat to the future viability of coral reef ecosystems (e.g., Hoegh-Guldberg *et al*., 2007; Riegl *et al*., 2009; Veron *et al*., 2009; Glynn, 2012), with studies linking widespread bleaching events to increasing sea surface temperatures (SSTs; Hoegh-Guldberg, 1999). There is concern that the growing frequency and severity of mass bleaching episodes may lead to species composition shifts and functional collapse in coral reefs in the near future (Hughes *et al*., 2003; Baker *et al*., 2008; Glynn, 2012; van Hooidonk *et al*., 2013), although this is likely to be strongly influenced by the extent of other anthropogenic stresses and the corals’ capacity for thermal adaptation (e.g., Baskett *et al*., 2009; Kennedy *et al*., 2013). In contrast, global warming also has the potential to improve currently marginal environmental conditions and extend the range of tropical coral reefs into higher latitudes (e.g., Precht & Aronson, 2004; Yamano *et al*., 2011), as demonstrated in the fossil record in response to warmer geological periods (e.g., Lighty *et al*., 1978; Veron, 1992; Precht & Aronson, 2004; Greenstein & Pandolfi, 2008; Woodroffe *et al*., 2010; Kiessling *et al*., 2012). If expansion is not limited by additional factors, higher latitude reefs could provide ‘refuges’ for many coral reef species as increasing temperatures cause ecosystem failure at low-latitude reef sites (e.g., Zamagni *et al*., 2012; Halfar *et al*., 2005; Vargas-Ángel *et al*., 2003; Glynn, 1996; though see, e.g., Lybolt *et al*., 2011).

A second global environmental pressure associated with rising atmospheric CO_2_ – ‘ocean acidification’ – complicates the temperature-only picture of a shift of suitable habitats to high latitudes and contraction in the tropics. Ocean acidification is the chemical consequence of the excess dissolution of CO_2_ derived from fossil fuel combustion in surface seawater (Turley *et al*., 2010). In addition to lower seawater pH, anthropogenic ocean acidification is also characterized by a reduction in the saturation state of calcium carbonate (Hönisch *et al*., 2012). Corals precipitate skeletons of the CaCO_3_ polymorph aragonite, and declining aragonite saturation (Ω_Arag_) increases the metabolic cost of skeletogenesis. Ocean acidification may thus lead to weaker reef structures that are more susceptible to erosion and competitive advantages for bioeroding organisms (Kleypas *et al*., 1999b; Smith & Buddemeier, 1992; e.g., Manzello *et al*., 2008). On the basis of this parameter (aragonite saturation) alone, it has been noted that for atmospheric CO_2_ concentrations beyond ca. 550 ppm, the expected open ocean surface Ω_Arag_ value for most reef locations will be below that associated with the limits of coral distributions today (ca. 3.25; Cao & Caldeira, 2008; Hoegh-Guldberg *et al*., 2007). This has raised concerns over the corals’ ability to maintain reef structures, pushing the view that declining aragonite saturation represents a pressing threat (e.g., Cao & Caldeira, 2008; Silverman *et al*., 2009; Ricke *et al*., 2013). However, the actual effect of increased acidification on calcification rates is complex and species dependent (Chan & Connolly, 2013; Kroeker *et al*., 2013), with many scleractinian corals being capable of buffering their internal pH for instance (e.g., Krief *et al*., 2010; McCulloch *et al*., 2012). The present-day relationship between coral reef locations and aragonite saturation is hence very likely modulated by other environmental factors such as temperature and nutrient availability (e.g., Cohen & Holcomb, 2009; Chauvin *et al*., 2011), meaning that habitat suitability implications cannot be drawn from future aragonite saturation changes in isolation.

With continuing fossil fuel CO_2_ emissions, these two main global marine environmental changes – surface warming and acidification, create a biogeographical tension, with warming tending to drive the zone of suitable coral reef habitat polewards, and decreasing saturation forcing it to contract toward the equator (Meissner *et al*., 2012; Yara *et al*., 2012). The net outcome is far from trivial, as the physiological responses of corals vary between regions, species, and even experimental manipulations, with interactions between stressors that are complex and nonlinear (e.g., Lough & Barnes, 1997; Silverman *et al*., 2007; Anthony *et al*., 2008; Chauvin *et al*., 2011; Edmunds *et al*., 2012). Despite a pressing need for a better understanding of the ongoing impact of environmental change on coral reef ecosystems (Pandolfi *et al*., 2011), few quantitative modeling studies have taken into account changes in SST and Ω_Arag_ simultaneously. Buddemeier *et al*. (2011) modeled the response to warming and acidification of eastern Caribbean reefs and projected that 95% coral cover would be lost by 2035, or delayed until 2065 if thermal tolerance of corals increases by 1–1.5 °C. Hoeke *et al*. (2011) found similar results for the Hawaiian region, with a precipitous decline in coral cover expected sometime between 2030 and 2050 unless corals are able to adapt to higher temperatures. They also predicted a temperature-driven increase in coral growth rates, particularly toward higher latitudes and despite the lower aragonite saturation levels there. Yara *et al*. (2012) explored changes in SST and Ω_Arag_ along the Japanese coastline. On the basis of a minimum Ω_Arag_ cutoff value of 3 (2.3) for tropical (temperate) corals, they found that a possible SST-driven range expansion into higher latitudes would be strongly limited by ocean acidification, and projected that all potential habitats in Japan might become unsuitable for tropical/subtropical coral reefs by 2040.

**Figure 1:**
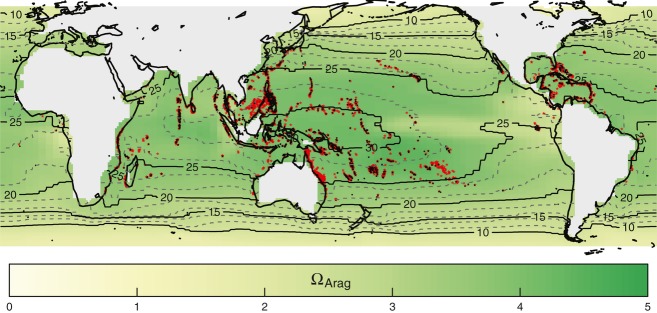
Current global distribution of coral reefs (red) according to ReefBase v2000 data set. The 1990 UVic-predicted fields for aragonite saturation (yellow–green color bar) and mean annual sea surface temperature (gray/black contour lines) used to train the Bioclimatic Envelope Models are also shown.

In this study, we assess global patterns of short-term range shifts in potential coral reef habitat under the spatially divergent stresses of ocean warming and acidification at the spatial scale of 1° × 1°. We employ a suite of statistical models based on the environmental factors thought to be limiting to the present equilibrium distribution of shallow-water coral reefs (Fig. 1, see Couce *et al*., 2012), perturbing them with Earth System Model projected future SST and aragonite saturation changes (the simulations used in Turley *et al*., 2010). We considered a range of potential future CO_2_ emissions scenarios, but focus here on the consequences of the ‘A2’ scenario (characterized by regionally oriented economic development and high population growth, expecting ca. 850 atmospheric CO_2_ by 2100). Significantly, the coral suitability models are not based on specific predetermined cutoff values for either Ω_Arag_ or SST, but instead seek to encapsulate the multidimensional climate envelope where the synergies between all relevant drivers favor the presence of coral reefs. Based on equilibrium relationships, these models do not incorporate information on coral responses or potential adaptation to dynamic environmental drivers, such as warming-induced bleaching and sea-level rise. Instead, the models identify where environmental conditions become adverse for coral reef presence based on the range of conditions in which they are currently found. The purpose of the study is thus not to predict the fate of existing coral reefs, but to identify the broad net impacts of warming and acidification on the potential future habitat suitability of these emblematic ecosystems.

## Materials and methods

### Habitat suitability models

Bioclimatic Envelope Modeling establishes relationships between environmental factors and the distribution of a species or ecosystem through statistical analysis. It defines an *n-*dimensional volume (or ‘envelope’) in the space of all relevant environmental factors where conditions are adequate for a species or ecosystem, identifying the relative significance of the different factors and the changes in environmental suitability across climatic gradients. We employed two different techniques: Maximum Entropy (MaxEnt; Phillips & Dudík, 2008; Phillips *et al*., 2006), and Boosted Regression Trees (BRT; De'ath, 2007; Friedman, 2001). Further details on these modeling approaches are provided in Table 1. Both MaxEnt and BRT are machine-learning techniques, able to handle nonlinear relationships and to take into account synergistic effects between the different factors affecting a species’ distribution. This flexible approach is advantageous for studying ecosystems because the tolerance limits of an ecosystem are influenced by contributions from many species and would be expected to give rise to more complicated relationships with climatic variables. MaxEnt is widely used in ecological studies, including the prediction of climate change impacts on a species or ecosystem's potential distribution (e.g., tree sparrows within North America, Graham *et al*., 2011a; sagebrush ecosystems in Nevada, Bradley, 2010; or invasive weeds species in Australia, Wilson *et al*., 2009). To date, BRT is less widely used, despite having comparable predictive capabilities (e.g., Elith *et al*., 2006; De'ath, 2007).

**Table 1:** Modelling techniques used in the study, model names used in article, and the list of environmental variables considered. See Materials and methods, Data S1 and Couce *et al*. (2012) for more details

Model Description	Model name	Variables
Boosted regression trees (BRT; Friedman, 2001) is a decision-tree-based statistical technique. A single tree is built by repeatedly splitting the data finding a cutoff value for one of the environmental variables so that the homogeneity of the resulting two groups is maximized. For BRT models, a sequence of trees (typically >1000) is produced, each grown on reweighted versions of the data, assigning ever-increasing weight to the cases misclassified by previous trees. The final prediction is obtained by the weighted average of predicted values across the sequence of trees.
	BRT_*OPT*_	27 environmental variables: Sea surface temperatures (SST; annual average, monthly maximum and minimum, annual range, standard deviation of monthly means, weekly maximum and minimum, and standard deviation of January and July means) Salinity (annual average, and monthly maximum and minimum) Nutrients (annual average phosphate and nitrate concentrations) Irradiance (annual average, and monthly maximum and minimum), attenuation coefficient at 490 nm wavelength (annual average), and depth of light penetration (annual average, and monthly maximum and minimum) Aragonite saturation (annual average Ω_Arag_) Dust level (annual average) Cyclone activity (30-year average) Current speed (annual average, and monthly maximum and minimum). For data sources see Couce *et al*. (2012)
BRT models were fitted in R, with version 1.6–3.1 of the *gbm* library (Ridgeway, 2007). Different values for the Tree Complexity (TC), Learning Rate (LR) and Bagging Fraction (BF) were considered, and the combination that minimized the predictive deviance was chosen in each case (for BRT_*OPT*_: TC = 10, LR = 0.01 and BF = 75; for BRT_*SIM*_: TC = 7, LR = 0.01 and BF = 50). See Couce *et al*. (2012) for more details.		
	BRT_*SIM*_	Five variables containing the most relevant information according to BRT_*OPT*_'s output: SST (annual average, monthly minimum, and weekly maximum) Depth of light penetration (monthly minimum) Irradiance (annual average) Plus aragonite saturation (annual average Ω_Arag_)
Maximum entropy modeling (MaxEnt; Phillips *et al*., 2006) is a presence-only technique for the prediction of species geographic distributions, based on the environmental conditions of sites of known occurrence. Assumes environmental factors act as constraints on the distribution of a species and that within those constraints, the species will occupy the available habitat in a way that maximizes entropy.
	MaxEnt_*OPT*_	Same variable set as BRT_*OPT*_
Our models were developed with Maxent version 3.3.3e, with default values for the convergence threshold (10^−5^) and maximum number of iterations (500).	MaxEnt_*SIM*_	Same variable set as BRT_*SIM*_

All models were trained with coral location data from ‘ReefBase’ (version 2000; http://www.reefbase.org; Vergara *et al*., 2000). This database was chosen for consistency and ease of comparison with previous studies: Kleypas *et al*. (1999a), Couce *et al*. (2012, 2013). At the 1° × 1° spatial resolution of our study, previous testing has shown the results are insensitive to the reef location data set used. For example, BRT and MaxEnt models developed using the UNEP-WCMC compilation (http://data.unep-wcmc.org/datasets/13; UNEP-WCMC, WorldFish Centre, WRI, TNC, 2010; IMaRS-USF, IRD, 2005; IMaRS-USF, 2005) replicated model performance and environmental variable relevance with insignificant differences to the equivalent ReefBase-trained models (E. Couce, unpublished data).

The optimal ‘OPT’ models were trained with a complete suite of relevant environmental fields identified in Couce *et al*., 2012 (a total of 27 parameters; these are the MaxEnt_*OPT*_ and BRT_*OPT*_ models), whereas the simple ‘SIM’ models were trained with a reduced subset of 6 of the most significant variables (the MaxEnt_*SIM*_ and BRT_*SIM*_ models) – see Table 1 for complete variable list. Annual average SST and Ω_Arag_ values were obtained from climate and marine carbon cycle projections made using the University of Victoria (UVic) Earth System Model (Weaver *et al*., 2001), while most of the remaining variables were observationally based. The UVic mean annual data were interpolated to the 1° × 1° resolution of the reef suitability models using the Inverse Distance Weighted method (2nd degree). Maximum and minimum SST values were computed by adding observed present-day anomalies to UVic 1990 mean SST. Additional information and analysis of the model development, data sources, and variables’ contribution to predictions are available in the Supporting Data S1 and is described in further detail in Couce *et al*. (2012).

### Grid and mask

All fields, including coral reef presence data, were mapped onto a 1° × 1° global grid between −60° and 60° latitude. The suitability models were trained on a shallow water mask established as the grid cells with regions shallow enough for adequate light penetration. The shallowest depth of a grid cell was determined from 30″ resolution SRTM30 Plus bathymetry data (Becker *et al*., 2009). The mask was defined as the grid cells for which the shallowest point fell within twice the mean annual depth of penetration of light (*Z*), estimated after Kleypas *et al*. (1999a): 

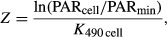
 where PAR_cell_ was the monthly mean Photosynthetically Active Radiation (PAR) using data from ISCCP (Bishop & Rossow, 1991; Bishop *et al*., 1997), PAR_min_ was 125 dWm^−2^ (to encapsulate mesophotic reefs; Gattuso *et al*., 2006) and *K*_490cell_ was the monthly mean diffuse attenuation coefficient of light (wavelength 490 nm) obtained from Globcolour (2008) satellite measurements. In addition, the study region was further constrained to the open-ocean areas covered by UVic projections (i.e., excluding the Red Sea and Arabian Gulf; Fig. S1.1 in Data S1). The environmental fields were extrapolated 1° shoreward by linear average of nearest neighbors to cover near-shore coral reef locations. This process resulted in a total of 32 582 oceanic grid cells, 4002 of which were located within the shallow-water mask. A total of 1124 cells contained at least one entry from the ReefBase v2000 data set (Fig. 1) and were designated ‘presence’ cells.

### Future projections

Future habitat suitability was computed by applying the Bioclimatic Envelope Models to UVic-generated projections for annual average SST and aragonite saturation under the IPCC TAR scenarios A2, B1, and A1B. Future maximum and minimum SST values were computed by adding observed present anomalies to UVic mean SST data (i.e., assuming future variability will remain the same). All other environmental fields were kept fixed at present values. Projections were then generated at 10-year intervals from 2010 to 2070.

To isolate the impacts of warming and ocean acidification, changes in SST variables and Ω_Arag_ were considered first separately and then in combination, while all other environmental variables were kept constant at modern observed values. Future projections can involve novel environmental conditions, exceeding the values of the models’ training range. Understanding the way in which the model deals with these data is critical for the correct interpretation of the results. The four models used in this study (Table 1) extrapolate by maintaining a constant response outside the training range (i.e., treating variables exceeding the values present in the training range as if they remained at the limit of the training range; see Data S2 for a detailed discussion on extrapolating to novel conditions and the impact on the predictions). In the results presented here, we explicitly indicate regions with novel SST or Ω_Arag_ conditions where the method of extrapolation significantly affects the projections and statistical relationships observed in training may no longer hold. These regions were defined as areas where MaxEnt_*OPT*_'s projected suitability was found to vary by 0.1 or more when extrapolation was carried out using a different method (i.e., by simple linear extrapolation of the model's response curves to each climatic variable, instead of maintaining a constant response). The 2070 cutoff for future projections was chosen because over 10% of coral reef cells are out of training range by this date.

### Model evaluation

Model performance on 1990 training data was tested via Receiver Operating Characteristic curves (ROC curves; Peterson *et al*., 1954) and Area-Under-the-Curve scores (AUC; Swets, 1988; Fig. S1.2 in Data S1). To assess the models’ predictive ability we considered published reports of fossil evidence from past warm geological periods and reports of present-day range expansion. We also explored whether rapid changes in equilibrium habitat suitability were correlated with areas where conditions have already been identified as challenging for existing coral reefs as evidenced by recent bleaching episodes. The data set of bleaching events was obtained from ReefBase (http://www.reefbase.org); it was originally developed by the WCMC and the WorldFish Center and subsequently extended to include published records and reports of personal observations. The changes in suitability between 2010 and training (1990) conditions on cells where bleaching episodes had been reported (between 2008 and 2012) were compared with the changes predicted in all reef presence cells; distributions and p-values of the two populations were compared via a Welsh's *t*-test, after testing the data for colinearity.

## Results

### Impact of future surface warming

**Figure 2:**
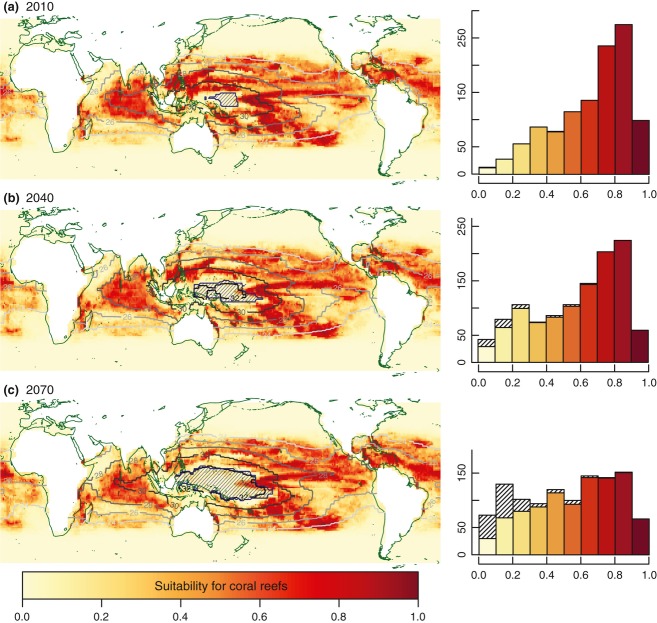
Predicted changes in suitability for coral reef ecosystems under the A2 scenario for future change in sea surface temperature (SST) variables only; 2010 (a), 2040 (b) and 2070 (c). Histograms show predicted suitability values for the 1124 presence sites of the study grid (i.e., cells currently with reefs according to the ReefBase v2000 data). All environmental fields apart from SST variables were kept constant at their present values (used in model training). Areas where projections are less reliable (i.e., SST out of training range and MaxEnt clamping value >0.1; see Data S2) are indicated by the hatched pattern, in both maps and histograms. The figure shows BRT_*OPT*_ model's results (see figure S3.1 in Data S3 for MaxEnt_*OPT*_'s projections).

Considering changes in SST variables only, sea surface warming has a marked impact on habitat suitability for coral reef ecosystems in currently favorable areas (defined as suitability ≥0.5), especially in the central Indo-Pacific region (Fig. 2). The percentage of presence cells (those currently with reefs or nonreef coral communities) for which the model is predicting suitability above a value of 0.5 falls from 77% in 2010 to 66% in 2040 and 54% by 2070 (BRT_*OPT*_ projections). This reduction is particularly pronounced in the Western Pacific Warm Pool (WPWP) region, affecting the Coral Triangle and some of the most biodiverse coral reef regions in the world. Suitability elsewhere either declines slightly (e.g., most of the Indian Ocean), or slightly increases (e.g., some areas of the Atlantic, including the Caribbean, and high latitudes). By 2070, SST conditions move out of training range for a large fraction of the Indo-Pacific region, with the area of less reliable model projection (as defined in ‘Materials and methods’) affecting up to ca. 13% of coral reef cells under the A2 scenario (hashed areas in Fig. 2 maps and histograms). If areas requiring extrapolation into novel conditions are excluded from the analysis, the observed patterns of change to 2070 remains robust (e.g., the decrease in habitat suitability among presence cells in the histograms in Fig. 2 is still evident).

### Impact of future ocean acidification

**Figure 3:**
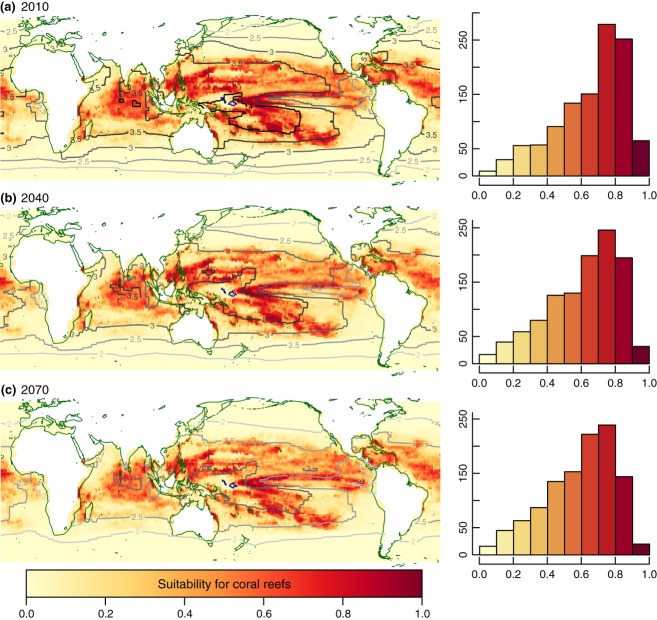
Predicted changes in suitability for coral reef ecosystems under the A2 scenario for future change in aragonite saturation only; 2010 (a), 2040 (b) and 2070 (c). Histograms show predicted suitability values for the 1124 presence sites of the study grid (i.e., cells currently with reefs according to the ReefBase v2000 data). All environmental fields apart from Ω_Arag_ were kept constant at their present values (used in model training). Unlike the SST results (Fig. 2), changes in Ω_Arag_ do not give rise to extrapolation uncertainties by producing novel conditions. The figure shows BRT_*OPT*_ results (see figure S3.2 in Data S3 for MaxEnt_*OPT*_ projections).

A reduction of surface Ω_Arag_ – while maintaining all other variables fixed at present values – leads to a decline in suitability for all areas currently favorable to coral reef formation (Fig. 3). However, the reduction is more subdued than that due to future SSTs (Fig. 2). For instance, whereas the projected SST rise caused the average suitability of presence cells to fall by 17%, from 0.69 in training (1990) conditions to 0.52 in 2070, the projected change in Ω_Arag_ over the same period caused a decrease of 10%, to an average of 0.59 (BRT_*OPT*_). The projected decline is also more uniformly distributed globally (Fig. 3) in contrast to the more extreme pattern of warming-induced impact. However, some geographical differences are still apparent, with regions most affected by increased acidification corresponding to off-equatorial zones in the Pacific, adjacent to the WPWP zone most affected by warming, and to a lesser degree in localized regions of the central Indian Ocean and the Caribbean. Unlike SST, changing Ω_Arag_ alone does not lead to extrapolation uncertainties (Fig. 3), as the areas with novel Ω_Arag_ conditions are confined to high latitudes, well beyond the present-day latitudinal range of scleractinian coral reefs.

### Synergistic impacts of warming and acidification

**Figure 4:**
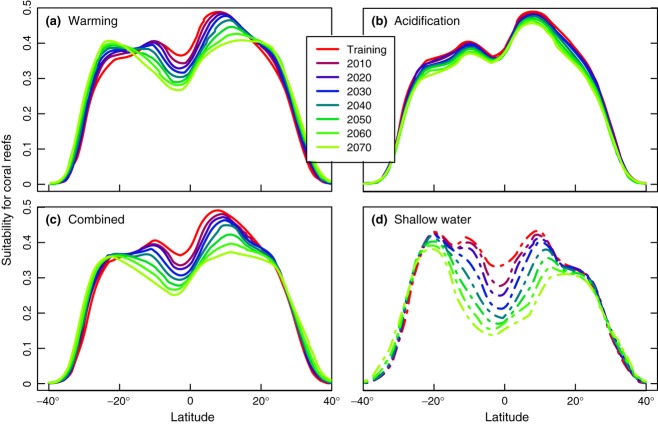
Predicted suitability of coral reef presence as a function of latitude, when changing sea surface temperature variables only (a), aragonite saturation only (b) and both factors simultaneously, for the entire ocean (c) and restricted to shallow waters (d). Different colors show variations over time under the A2 scenario. All figures have been made averaging the modeled suitability values in 5° latitudinal bands, and show MaxEnt_*OPT*_ model's results (see figure S3.3 in Data S3 for the equivalent BRT_*OPT*_ projections).

On a zonally averaged basis, ocean acidification reduces the environmental suitability for reef presence across all latitudes, but maximal influence is in the off-equatorial zone (ca. 5° to 20° in both hemispheres, Fig. 4a). The projected rise in SST significantly reduces coral reef suitability for all latitudes between 15°S and 15°N, while driving slight increases at latitudes above ca. 20° (Fig. 4b). With simultaneous changes in SST and Ω_Arag_, the suitability for coral reef presence drops across all latitudes between 20°S and 20°N (Fig. 4c). This reduction is more extreme when the analysis is restricted to the grid cells with substrate within the euphotic zone adequate for reef formation (Fig. 4d). The SST-driven expansion at latitudes above 20° still persists, although it is restricted by the countereffect of acidification.

**Figure 5:**
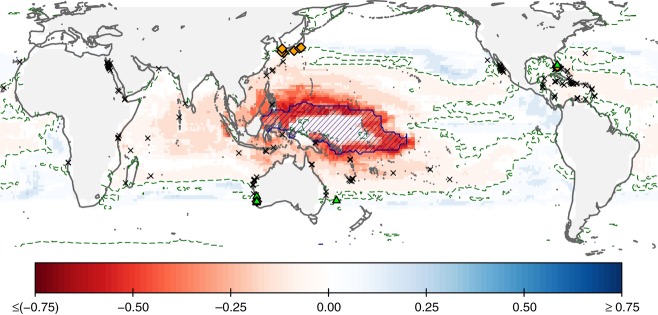
Projected change in suitability for coral reef ecosystems between 1990 and 2070 under the A2 scenario, considering simultaneous changes in aragonite saturation and surface ocean temperature. The projected response has been averaged across both ‘OPT’ models and the MaxEnt_*SIM*_ model (models defined in Table 1; BRT_*SIM*_'s results were excluded from the average, as discussed in the text and Data S3). Green dashed line indicates null projected change, whereas the hatched pattern identifies areas with novel conditions where projections are less reliable, due to a significant impact of the chosen extrapolation method (as explained in Methods). The sites of past (green triangles) and present (orange diamonds) range expansion of coral are also indicated, based on data from Lighty *et al*. (1978), Veron (1992), Marsh (1993), Vargas-Ángel *et al*. (2003), Greenstein & Pandolfi (2008), Woodroffe *et al*. (2010), and Yamano *et al*. (2011). Black crosses indicate coral reef distribution during the last interglacial period (data from Kiessling *et al*., 2012).

**Figure 6:**
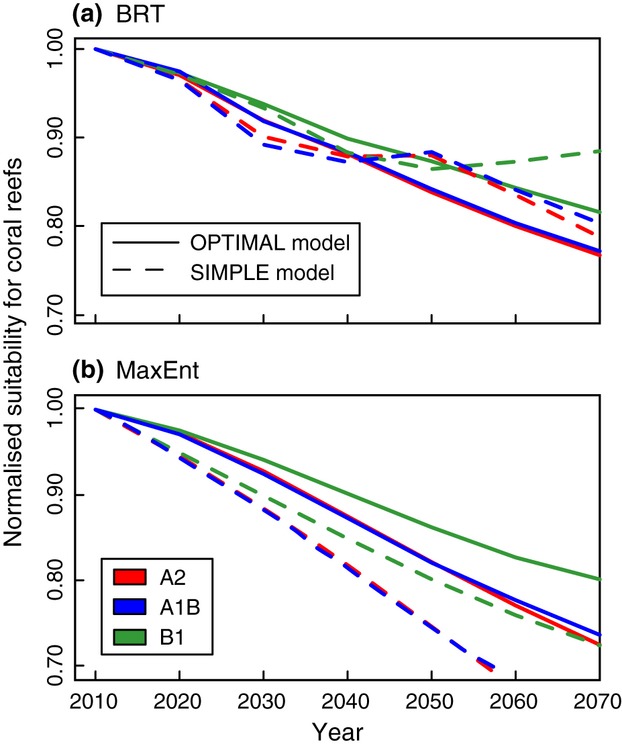
Average global suitability for coral reef presence in shallow water areas over time for boosted regression trees (a) and MaxEnt (b) models. Color of the line refers to the CO_2_ emission scenario, with red, blue, and green for A2, A1B, and B1, respectively, while the pattern of the line represents the model, with continuous lines for ‘OPT’, and dashed for ‘SIM’ models. Suitability values have been normalized to 2010 average.

The zonal averaged changes (Fig. 4) conceal a more heterogeneous geographical response, with the 2070 projections forecasting decreased suitability for coral reef ecosystems in nearly all reef areas (including the Caribbean, GBR, and Coral Triangle region) and with the greatest decline in suitability taking place in the western Pacific (Fig. 5). All four models project a 27–30% decline within shallow waters over the next 60 years (Fig. 6). The three CO_2_ emission scenarios considered (A2, A1B, B1) produce very similar projected changes in suitability, corresponding to the consistent levels of warming to 2070 expected across all scenarios (IPCC, 2007).

### Model performance, comparison with fossil data and recent bleaching episodes

**Figure 7:**
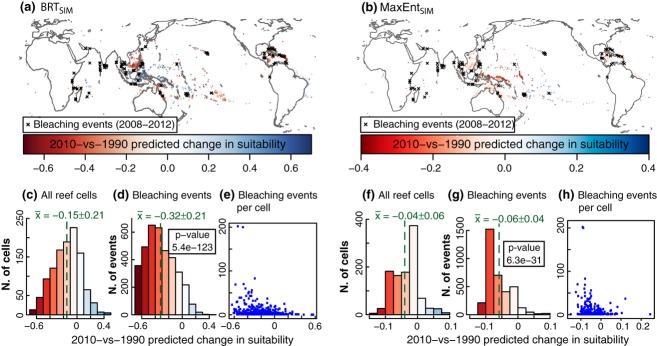
Changes in suitability for coral reef ecosystems between 1990 and 2010 predicted by BRT_*SIM*_ (left) and MaxEnt_*SIM*_ (right) and the point location of over 3500 bleaching events taking place between 2008 and 2012 according to the ReefBase database. The changes in suitability assigned to grid cells currently with reefs (c, f) provide the baseline to which those corresponding to the bleaching events’ locations (d, g) should be compared (in figures c, d, f and g the mean and standard deviation are indicated). The p-values from a Welsh's *t*-test comparing the suitability distribution of bleaching events and that of all reef cells is also shown in d and g. Figures e and h illustrate the number of bleaching events within a cell as a function of the change in suitability assigned to that cell.

All models performed adequately on training data, with average AUC scores close to 0.9 when evaluated on a random 25% subset data that had been excluded for model training (Fig. S1.2 in Supporting Data S1). The simple MaxEnt_*SIM*_ model, based on a reduced set of climatic variables, showed the poorest performance in ROC space, with an AUC score of 0.84 ± 0. 01. There is a clear correspondence between the changes in suitability predicted for present-day (2010) by the ‘SIM’ versions of the models and the locations of over 3500 reported bleaching episodes from 2008 to 2012 (Fig. 7). However, this correlation was not present in the forecasts obtained with the full ‘OPT’ models. In general, there was good agreement between forecasts obtained with the different models (Data S3), with the exception of BRT_*SIM*_.

Sites of observed range expansion and fossil evidence from warmer periods in the past (based on data from Kiessling *et al*., 2012; Woodroffe *et al*., 2010; Greenstein & Pandolfi, 2008; Veron, 1992; Lighty *et al*., 1978) are consistent with the areas showing improved environmental suitability in our future projections (Fig. 5). In addition, the SST-driven decline projected at low latitudes is consistent with the equatorial decline described by Kiessling *et al*. (2012) from analysis of locations of coral fossil records from the last interglacial period.

## Discussion

### Isolating the impacts of warming and acidification

Areas where projected coral reef habitat suitability is most critically degraded by ocean surface warming (Fig. 2) correspond to areas with the highest mean annual SSTs today (Fig. 1), and also map onto regions identified as being particularly susceptible to future coral bleaching (e.g., Guinotte *et al*., 2003; Donner *et al*., 2005; van Hooidonk *et al*., 2013). We find that the projected decrease in suitability is most strongly associated with the weekly maximum – and to a lesser degree, minimum – SST variable, and very high values (>32 °C) are linked to marked declines (see Figs. S1.7–S1.8 in Data S1 and Fig. S2.5 in Data S2). Both BRT and MaxEnt statistical modeling approaches assign a relatively high significance to maximum weekly SST; in 10 randomly generated versions of MaxEnt_*OPT*_/BRT_*OPT*_, this parameter ranked 5th/8th of a total of 27 parameters in average relative contribution to model output (however, correlations between variables do make it difficult to clearly establish hierarchy; Couce *et al*., 2012). The importance placed by the Bioclimatic Envelope Models on SST variables in general, and the negative contribution to modeled suitability at the higher end of the SST range, indicates that the global data set used for model training contains sites where present-day coral reef distribution is already limited by an upper thermal threshold. Although equivalent maximum SSTs are tolerated by Red Sea and Arabian Gulf coral reefs, these sites could not be included in the training data set and analysis because it was not possible to simulate conditions using the UVic model in these enclosed seas. It has been suggested that Red Sea and Arabian Gulf coral are potentially conditioned to extreme SSTs by very high SST variability (Ateweberhan & McClanahan, 2010), however, this is not a characteristic shared with the equatorial regions projected to decline in habitat suitability (Lough, 2012). Adding support to our model projections of an equatorial retraction, in addition to a poleward expansion, is the matching distribution range shift identified in fossil records of coral distribution during the last interglacial period, when the global average temperature exceeded that of today (Kiessling *et al*., 2012).

The areas most affected by reductions in Ω_Arag_ levels correspond to off-equatorial latitudes, particularly in the western Pacific (Fig. 3 and 4b). Generally highly stratified, therefore with little buffering by mixing with subsurface waters, these regions will tend to experience the strongest degree of saturation decline. In addition, these are slightly cooler waters, where other environmental factors, including Ω_Arag_, are likely to out-weigh temperature variables in terms of importance. For instance, Cooper *et al*. (2012) argued that, at present, SST was far more significant than Ω_Arag_ to explain long-term changes in calcification rates for massive *Porites* colonies off Western Australia, whereas the GBR has been identified, in modeling studies (McNeil *et al*., 2004) and skeletal measurements in massive *Porites* colonies (e.g., Cooper *et al*., 2008; De'ath *et al*., 2009), as a region already exceeding the thermal optima for coral calcification and sensitive to reduced Ω_Arag_.

In contrast, our results suggest higher latitude sites (e.g., southern GBR, southern Japan) and upwelling regions (e.g., East Pacific) will be relatively little affected by the acidification resulting from the projected increases in atmospheric CO_2_. The suitability for reef presence at 1990 levels is maintained in these locations up until 2070 across the majority of the models (Fig. 3). These areas experience the lowest Ω_Arag_ levels at which reefs are found today and are considered marginal for reef formation (e.g., Smith, 1992; Kleypas *et al*., 1999a; Glynn *et al*., 2007; Manzello *et al*., 2008) due to low calcification and cementation rates, which are finely balanced with erosion processes (Glynn, 1976; Cortes, 1997). Previous studies (e.g., Hoegh-Guldberg *et al*., 2007; Cao & Caldeira, 2008; Manzello, 2010) have identified coral reefs from higher latitudes and upwelling regions as the most sensitive to future reductions in Ω_Arag_. However, statistical modeling has indicated that SST variables and light availability may be stronger determinants of coral reef presence at these marginal sites than Ω_Arag_ (Couce *et al*., 2012). Our future projections reflect this balance of controls. In addition, upwelling of deeper waters, isolated from the atmosphere, leads to a substantial degree of pCO_2_ disequilibrium between ocean surface and atmosphere and may hence provide some buffering against ocean acidification in regions such as the Eastern Pacific. Fabricius *et al*. (2011) observed changes in coral reef communities along a natural pH gradient on volcanic seeps at Papua New Guinea, and found that despite significant losses in species composition and biodiversity, coral cover remained constant for acidification levels comparable to what is expected by 2070 under the A2 scenario. This was achieved through a transition from branching species such as *Acropora* sp. toward predominance of more massive *Porites* sp. corals, whose calcification rates were less affected. Such shifts in species composition could have significant implications for ecosystem services; however, this study models coral reef ecosystems as a single entity, and is not sensitive to changes in species composition or biodiversity losses.

### Synergistic impacts of warming and acidification

Under the combined influences of ocean surface warming and acidification, habitat suitability for coral reef ecosystems declines across the latitudinal band between 20°N and 20°S, affecting significant reef areas such as the Caribbean, GBR, and Coral Triangle region (Fig. 4c and 5). Areas where shallow water substrate is available are particularly susceptible to the impacts of warming and acidification (e.g., in Fig. 4d, modeled suitability in shallow equatorial regions more than halves by 2070). Although a marginal improvement at higher latitudes is projected by 2070, range expansion is constrained by availability of shallow waters where benthic substrate is present within light penetration depths (Fig. 5). Range expansion is also projected onto areas at the extreme end of oceanographic circulation pathways along the western boundaries, where the most depauperate reefs are found today (e.g., Glynn *et al*., 2007; Macintyre, 2003; Veron & Minchin, 1992; but see Thomson & Frisch, 2010). Coral reef expansion to higher latitude sites with improved conditions will, therefore, depend on larval influx, introducing a likely limitation for sites situated away from current transport such as the south-eastern Pacific (Wood *et al*., 2013). The rate of projected range expansion of suitable environmental conditions for coral reef presence varies between models. The MaxEnt_*OPT*_ model predicts a moderate (ca. 5 km per decade) expansion at present, accelerating to 30–45 km per decade by 2070 (Fig. 4d; calculated as rate at which curve edges intersects a horizontal line at 0.2). In contrast, BRT_*OPT*_ projects an initial global shrinkage under the effects of acidification (at ca. 25 km per decade), with increasingly rapid expansion from 2020–2030 as the effect of rising SST becomes dominant (up to 70–80 km per decade by 2070). For comparison, Burrows *et al*. (2011) found the average poleward speed of surface isotherm movement in the oceans (50°S to 80°N) over the last 50 years was 27.5 km per decade. As already stated, our coral reef range expansion rate estimates do not take into account connectivity limitations related to the dispersal of coral larvae by ocean currents, and thus follow the thermal shift tempered by ocean acidification.

The combined impacts of increasing SST and acidification have little impact on environmental suitability for coral reef presence in the eastern Pacific, south east Atlantic, and the north Brazilian coast over the coming decades. These are recognized as marginal reef sites (e.g., Smith, 1992; Kleypas *et al*., 1999a; Glynn *et al*., 2007; Manzello *et al*., 2008); however, the decline of Ω_Arag_ is relatively slow in these predominantly upwelling regions, average SST is within tolerance levels, and further increases in SST might actually be advantageous for reef formation. The low Ω_Arag_ values in the eastern Pacific are not a limiting factor according to any of our statistical models, which instead assign constant, or at times increasing, suitability values to areas with Ω_Arag_ values falling below 2.2–2.3. While these levels of Ω_Arag_ will obviously impact reef structure and the balance of carbonate accretion and dissolution (e.g., Kleypas *et al*., 1999b; Manzello *et al*., 2008), and is likely to affect species composition and biodiversity (e.g., Fabricius *et al*., 2011), the region remains suitable for reef presence in our models, potentially assisted by warming where temperatures were previously suboptimal. That conditions remain suitable does not necessarily imply that existing reefs will be able to cope with the changes anticipated by 2070. We have not considered future changes in SST variability in our analysis, and this factor could play a key role for coral reef survival in these marginal areas (Glynn & Colgan, 1992; Toth *et al*., 2012), which are ranked globally among the slowest in terms of recovering from disturbances (Graham *et al*., 2011b).

### Model evaluation and limitations

Empirical evidence is important to increasing our confidence in Bioclimate Envelope Models and projected distribution changes (Araújo *et al*., 2005) and the fossil coral record can provide a test for the models’ predictive ability. If SST is the stronger limiting factor for the presence of shallow coral reefs at high latitudes, we would expect fossil evidence of such an expansion into higher latitudes during warmer geological periods. In Fig. 5, we compare our 2070 predictions with relevant fossil records, including reports of extensive relic reef formations in high-latitude sites in Japan (Veron, 1992), Florida (Lighty *et al*., 1978), and Lord Howe Island (Woodroffe *et al*., 2010) dating from the Holocene, and along the Western Australian coast dating back to the Late Pleistocene (Greenstein & Pandolfi, 2008). Kiessling *et al*. (2012) documented both a range expansion and an equatorial retraction of coral reef distribution during the last interglacial period of the Pleistocene (ca. 125 000 years ago; Fig. 5), when average SST might have been about 1 °C higher than today (McKay *et al*., 2011). In addition, there is evidence supporting present-day range expansion. Yamano *et al*. (2011) report several tropical coral species expanding their range into higher latitudes along the coast of Japan, among them two *Acropora* sp. key for reef formation in the Indo-Pacific region. Veron (1992) also describes a high latitude fossil reef in Japan being re-colonized as a response to increasing temperature and Marsh (1993) reported *Acropora* sp. growing at a high-latitude site off Perth, Western Australia. In the Atlantic region, Vargas-Ángel *et al*. (2003) describe thickets of *Acropora cervicorvis* growing off the coast of Florida at higher latitudes than found previously. All these reports of past and present range expansion are consistent with our projections under current environmental change (Fig. 5).

Bioclimatic Envelope Models trained with equilibrium data are not suitable tools for the prediction of transient coral bleaching episodes. In particular, the models employed here have not been trained with the most relevant environmental variables (e.g., Degrees Heating Weeks or other short-term measures of thermal stress), or the location, date, and severity of bleaching episodes. Our projections correspond, instead, to the expected equilibrium response if conditions were maintained constant for sufficient time. Within these limitations, we wanted to establish a comparison of our predictions for the present-day with current observations. Coral bleaching reflects a stress response (Glynn, 1996), and degrading environmental conditions may lead to an increased number of bleaching episodes. To test this hypothesis, we compared the distribution of over 3500 recent bleaching events (observed between 2008 and 2012) with projected changes in suitability between model training conditions (1990) and the present-day (2010). We found bleaching episodes were more frequent in areas to which both MaxEnt_*SIM*_ and BRT_*SIM*_'s 2010 projections assign higher decreases in suitability, when compared to the average on presence cells (Fig. 7). This is particularly significant for the BRT ‘SIM’ model; however, no such correlation is present with projections by the ‘OPT’ models, developed from the full suite of 27 predictive variables. Models relying on a limited number of variables are more sensitive to any change in those variables, and thus the responses of the ‘SIM’ models generally amplify that of the ‘OPT’ models, which can help explain why the ‘SIM’ models correlate better to short-term variations. MaxEnt_*SIM*_ and BRT_*SIM*_ do however differ substantially in their predictions, especially in the WPWP region and surrounding areas, where the highest decrease in suitability is to be found according to MaxEnt_*SIM*_, but where BRT_*SIM*_ actually forecasts improved conditions. Few bleaching events are reported for the WPWP, but as it contains some of the least monitored coral sites in the world, this may be due to underreporting. BRT methods tend to overfit to training data, despite careful model development designed to minimize this (e.g., Elith *et al*., 2008), and models relying on a limited number of variables are more affected. Despite acceptable performance by BRT_*SIM*_ within training conditions (similar AUC scores to that of MaxEnt_*SIM*_; Fig. S1.2 in Data S1), overfitting makes its projections unstable, creating an oscillatory response to rising temperatures (e.g., Fig. S1.7) and leading to unreliable projections for anything but the smallest changes in environmental variables, including out-of-range conditions (e.g., the WPWP and surrounding areas in Fig. 7). For this reason, we chose to exclude BRT_*SIM*_ projections from the average 2070 change in suitability (Fig. 5). Interestingly, the same qualities that make BRT_*SIM*_ a poor long-term predictor may determine its strength in identifying areas that are currently experiencing thermal stress (Fig. 7). The MaxEnt technique is not as prone to overfitting, and we find strong agreement between projections by the ‘SIM’ and ‘OPT’ model versions. In addition, this issue is not present with the full ‘OPT’ version of the BRT model, which has a similar performance to both MaxEnt models (Data S3).

Caveats in the use of Bioclimatic Envelope Models to predict the effect of climate change on the distribution of a species include uncertainties associated with the modelling technique used (e.g., Thuiller, 2004; Lawler *et al*., 2006), migration limitations (reviewed in Thuiller *et al*., 2008), the method employed for out-of-range extrapolation (e.g., Webber *et al*., 2011), the availability of absence data or background selection (e.g., Elith *et al*., 2010), the spatial scale of the analysis (e.g., Pearson & Dawson, 2003), and the equilibrium hypothesis (e.g., Hirzel *et al*., 2001). Some of these caveats can be addressed by understanding the limitations of the results, which represent changes in equilibrium habitat suitability. Projections of the fate of existing coral reef ecosystems require the consideration of additional factors, such as local anthropogenic pressures and management, stress responses and recovery, bleaching, mortality, rates of sea-level rise, potential for adaptation, and migration capabilities among others. Instead, the Bioclimatic Envelope Models used here simply identify areas with future conditions similar to those where present-day coral reefs are found. In addition, the 1° × 1° spatial scale of the study may be too coarse to resolve many of the subtleties of the changing environmental conditions; however, this scale is typical of global projections from current climate models. Within those limitations, our results are informative, representing an initial estimation of the magnitude of the impact and providing a basis for future modelling work.

We employed two Bioclimatic Envelope Model approaches that make different use of absence data. MaxEnt is a presence-only technique, meaning it does not assume absence in areas where presence has not been established, unlike BRT. These two approaches have divergent responses to some of the limitations listed above, including performance in out-of-equilibrium situations (Hirzel *et al*., 2001) and dealing with incompleteness of the training database and/or absences due to factors other than climatic unsuitability (e.g., Pulliam, 2000). In addition, the development of models with two different levels of complexity – the ‘OPT’ and ‘SIM’ versions – helps establish the impact of variable selection in the model output and illustrate model-related performance issues, such as BRT_*SIM*_'s poor performance for long-term and out-of-equilibrium predictions. Overall, we find relatively high model agreement between presence-only MaxEnt and presence/absence BRT (for the ‘OPT*’* versions) and between the ‘OPT*’* and ‘SIM*’* model versions in the case of MaxEnt, particularly in areas projected to experience worsening conditions (Fig. 5 and Data S3). This, together with the congruence with the distribution of fossils from warmer geological times, increases our confidence in the predictions.

Despite their limitations, we note that the use of Bioclimatic Envelope techniques together with climate models output remains among the best tools in the study of species or ecosystem responses to changing conditions (e.g., Pearson & Dawson, 2003; Wiens *et al*., 2009). This study represents a significant advance over previous studies discussing the conflicting effects of warming and acidification because our models do not rely on specified thresholds for SST and Ω_Arag_ variables, but instead make simultaneous use of all relevant variables in the definition of an optimal climatic ‘envelope’ based only on the statistical analysis of the current coral reef distribution. This ‘envelope’ allows for the synergistic or antagonistic responses between variables that complicate physiological experimental results of thermal tolerance and ocean acidification studies.

Our main findings for future environmental suitability of coral reef ecosystems in all three CO_2_ emission scenarios considered are as follows: (i) range expansion at the high-latitude boundaries; (ii) no decreased suitability in currently marginal eastern Equatorial Pacific locations as well as in the Atlantic generally; and (iii) severe temperature-driven impacts in the WPWP and surrounding regions. The potential range expansion at high latitudes, however, may in many places be severely constrained by a lack of suitable benthic environment available for colonization and could additionally be affected by dispersal limitations. Currently, reefs in the Eastern Tropical Pacific will remain marginal, with increased warming offsetting the negative impacts of ocean acidification, while impacts in the suitability of the Atlantic basin as a whole may be minor. However, our models also forecast a significant overall decline in coral reef habitat suitability, with a decrease in suitability for coral reef ecosystems by 2070 of up to 30% in shallow water areas. We find that the decline, driven mainly by short-term SST maxima, is greatest around the WPWP region, and therefore would affect some of the most biodiverse coral reef regions. These results present important implications for future coral reef management, as they suggest that more emphasis should be placed in conservation efforts on marginal reefs as they are not necessarily a ‘lost cause’. They also suggest that coral reef presence is more likely to be preserved throughout much of the central and western Indian Ocean as well as the Atlantic, assuming other anthropogenic stresses are minimized.

## Supporting Information

**Data S1**. Further details on the Bioclimatic Envelope Models. Details on model training, shallow water mask used in the study, variable contributions, and model performance on training data. **Data S2**. Extrapolating to novel climates. Details on the method used to extrapolate for data outside training range, and discussion on its impacts on projections and the regions affected. **Data S3**. Analysis of model agreement. Alternative model results for all figures presented in the main document, and discussion on agreement between 2070 projections by all four models.
