# Supplementary material for: Future habitat suitability for coral reef ecosystems under global warming and ocean acidification
Source: Glob Chang Biol. 2013 Oct 8;19(12):3592–606. doi: 10.1111/gcb.12335 (PMC4028991; doi:10.1111/gcb.12335)
Supplement: Supplementary file 1 — Data S1. Further details on the Bioclimatic Envelope Models. Details on model training, shallow water mask used in the study, variable contributions, and model performance on training data. Data S2. Extrapolating to novel climates. Details on the method used to extrapolate for data outside training range, and discussion on its impacts on projections and the regions affected. Data S3. Analysis of model agreement. Alternative model results for all figures presented in the main document, and discussion on agreement between 2070 projections by all four models. [file gcb0019-3592-sd1.docx]

**SUPPORTING INFORMATION**

The additional Supporting Information for the online version of this article:

**Data S1: Further details on the bioclimatic envelope models**

Table S1.1: Variables’ contribution to each of the models.

Figure S1.1: Shallow water mask.

Figure S1.2: ROC curves and AUC scores for all four models.

Figure S1.3: Fitted values plots for *BRT_OPT_* model.

Figure S1.4: Fitted values plots for *MaxEnt_OPT_* model.

Figure S1.5: Fitted values plots for *BRT_SIM_* model.

Figure S1.6: Fitted values plots for *MaxEnt_SIM_* model.

Figure S1.7: Marginal response curves for *BRT_SIM_* model.

Figure S1.8: Marginal response curves for *MaxEnt_SIM_* model.

**Data S2: Extrapolating to novel climates**

Figure S2.1: Comparison of SST and Ω_Arag_ values in training and future projections.

Figure S2.2: High SST areas outside training range.

Figure S2.3: MESS map and most out-of-range variable.

Figure S2.4: *MaxEnt_OPT_* clamping map for 2070, A2.

Figure S2.5: SST response curves and different extrapolation methods.

Figure S2.6: *MaxEnt_OPT_*’s training prediction and projected change in 2070, A2, using different extrapolation methods.

**Data S3: Analysis of model agreement**

Figure S3.1: Alternative Figure 2 for the *MaxEnt_OPT_* model

Figure S3.2: Alternative Figure 3 for the *MaxEnt_OPT_* model

Figure S3.3: Alternative Figure 4 for the *BRT_OPT_* model

Figure S3.4: 2070 projections for each individual model

Figure S3.5: Direct cell-by-cell comparison of 2070 projected change

Figure S3.6: Fuzzy numerical ­­­­­­comparison maps for 2070 projected change

Figure S3.7: Sign-agreement map for 2070 projections.

**Data S1: Further details on the bioclimatic envelope models**

The bioclimatic envelope models were trained within a shallow water mask defined by bathymetry within the euphotic zone and the area covered by UVic projections (Fig. S1.1, more details on the mask can be found in the *Methods* section of the main manuscript). The definition of all variables used in the analysis and the relative contribution of each of them to the models’ output is listed in Table S1.1. For the exact definition of how the contribution is estimated see Friedman & Meulman ([2003](#_ENREF_5)) for BRT and Phillips ([2006](#_ENREF_7)) for MaxEnt. The values in Table S1.1 are misleading and need to be interpreted with caution due to a high level of correlation between many of the variables.

**Figure S1.1:** Shallow water mask (blue-grey) and cells containing reef and non-reef coral communities according to the ReefBase v2000 dataset (red).

Figure S1.2 shows the Receiver operating characteristic (ROC) curves for all four models of Table S1.1. A ROC curve provides a simple measure of model performance for binary classifier systems, expressed as the number of true positives versus false positives obtained by the model. The different curves show the performance of 10 different models, generated each time from a random choice of 75% of the data (training data) and tested on the remaining 25% (evaluation data). The Area Under the Curve (AUC) scores indicated in the figure correspond to the average value and standard deviation from those 10 models. As a reference, Swets ([1988](#_ENREF_10)) considered AUC scores above 0.9 indicated ‘highly accurate’ models whereas scores below 0.7 showed poor performance.

Figures S1.3-S1.6 show the models’ predicted suitability values for training conditions as a function of the values of each of the variables. This type of plot can provide a guide of the way the model is making use of the variable, when combined with the contribution score listed in Table S1.1. For highly correlated variables they are often easier to interpret than response curves (only shown for SIMPLE models, Figs. S1.7 and S1.8).

| **Variable code** | **Variable explanation** | **Relative contribution (%)** | | | |
| --- | --- | --- | --- | --- | --- |
|  |  | ***BRT_OPT_*** | ***MaxEnt_OPT_*** | ***BRT_SIM_*** | ***MaxEnt_SIM_*** |
| SST_month_min | Min. monthly SST mean | 13.4 | 2.0 | 27.2 | 8.7 |
| SST_mean | Average of monthly SST means | 12.0 | 57.5 | 21.1 | 69.7 |
| Z_month_min | Min. monthly mean of light penetration depth | 6.4 | 4.2 | 17.0 | 6.7 |
| Irr_mean | Mean annual irradiance | 5.0 | 2.4 | 11.8 | 2.8 |
| SST_week_max | Max. weekly SST mean | 5.0 | 2.3 | 10.5 | 8.7 |
| Ω_Arag_ | Mean annual aragonite saturation | 3.0 | 3.1 | 12.5 | 3.4 |
| NO3 | Mean annual nitrate concentration | 4.8 | 1.7 |  |  |
| Z_month_max | Max. monthly mean of light penetration depth | 4.2 | 2.4 |  |  |
| Irr_month_max | Max. monthly irradiance means | 3.9 | 1.3 |  |  |
| Irr_month_min | Min. monthly irradiance means | 3.8 | 0.5 |  |  |
| JanSST_std | SD of mean January SST in 1982-2009 | 3.3 | 0.5 |  |  |
| PO4 | Mean annual phosphate concentration | 3.3 | 1.9 |  |  |
| Sal_mean | Average of monthly salinity means | 3.2 | 0.9 |  |  |
| Sal_month_min | Min. monthly salinity | 3.0 | 2.5 |  |  |
| SST_month_max | Max. monthly SST mean | 2.9 | 6.0 |  |  |
| Dust | Mean annual dust concentration | 2.8 | 1.1 |  |  |
| SST_std | SD of monthly SST means | 2.5 | 0.4 |  |  |
| JulSST_std | SD of mean July SST in 1982-2009 | 2.4 | 0.3 |  |  |
| SST_range | Difference between max. and min. monthly SST mean | 2.3 | 0.6 |  |  |
| Current_mean | Average of monthly current speed means | 1.9 | 0.1 |  |  |
| SST_week_min | Min. weekly SST mean | 1.8 | 6.5 |  |  |
| Sal_month_max | Max. monthly salinity | 1.8 | 0.7 |  |  |
| Z_mean | Average of monthly means of light penetration depths | 1.7 | 0.1 |  |  |
| Storm | 30-year average cyclone activity | 1.6 | 0.6 |  |  |
| Current_min | Min. monthly current speed | 1.5 | 0.1 |  |  |
| Current_max | Max. monthly current speed | 1.3 | 0.2 |  |  |
| K490 | Mean annual 490nm attenuation coefficient | 1.3 | 0.5 |  |  |

**Table S1.1:** Variable definitions and relative contributions to each model. (WARNING: correlated explanatory variables’ contributions are difficult to untangle, and for them the values provided here should be considered unreliable).


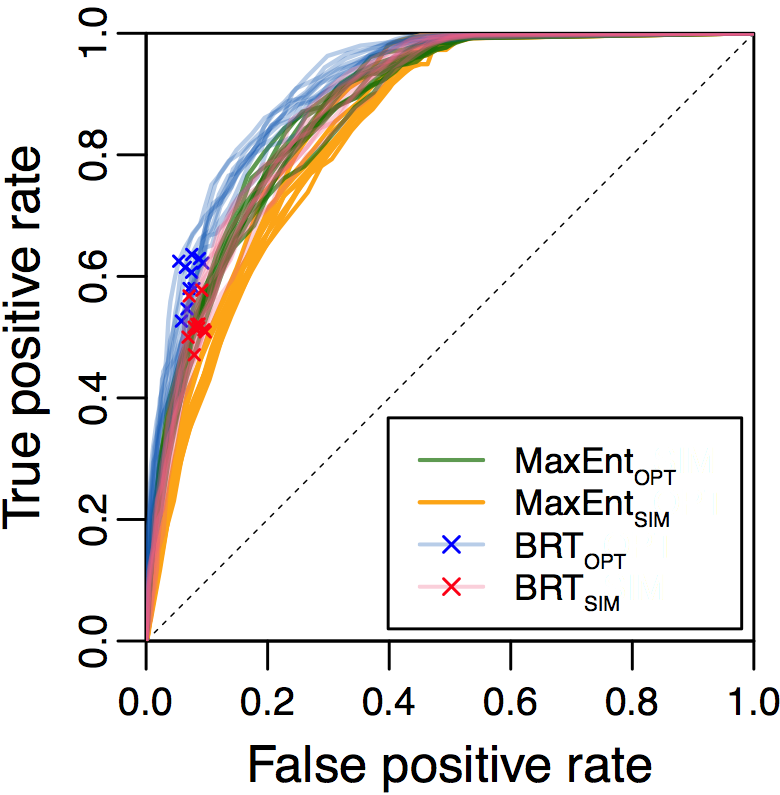


| **Model** | **AUC score** |
| --- | --- |
| MaxEnt_OPT_ | 0.87 ± 0.01 |
| MaxEnt_SIM_ | 0.84 ± 0.01 |
| BRT_OPT_ | 0.91 ± 0.01 |
| BRT_SIM_ | 0.87 ± 0.01 |

**Figure S1.2:** Receiver operating characteristic (ROC) curves for the four models obtained from the variables indicated in Table S1.1 (left), and corresponding Area-Under-the-Curve (AUC) scores (right). For each model there are 10 curves, each corresponding to the performance of a different model, trained from a random 75% subset of the data, and tested on the remaining 25%. The plot describes the model’s true positives versus false negatives as the discrimination threshold (the cut-off value from which a cell is considered presence or absence) is allowed to change. For the two BRT models the point in ROC-space corresponding to a discrimination threshold of 0.5 (50% suitability for coral reefs) has been indicated with an ‘x’. The AUC scores have been obtained from the mean and standard deviation of the 10 randomly-generated models showed in the ROC plot.

**
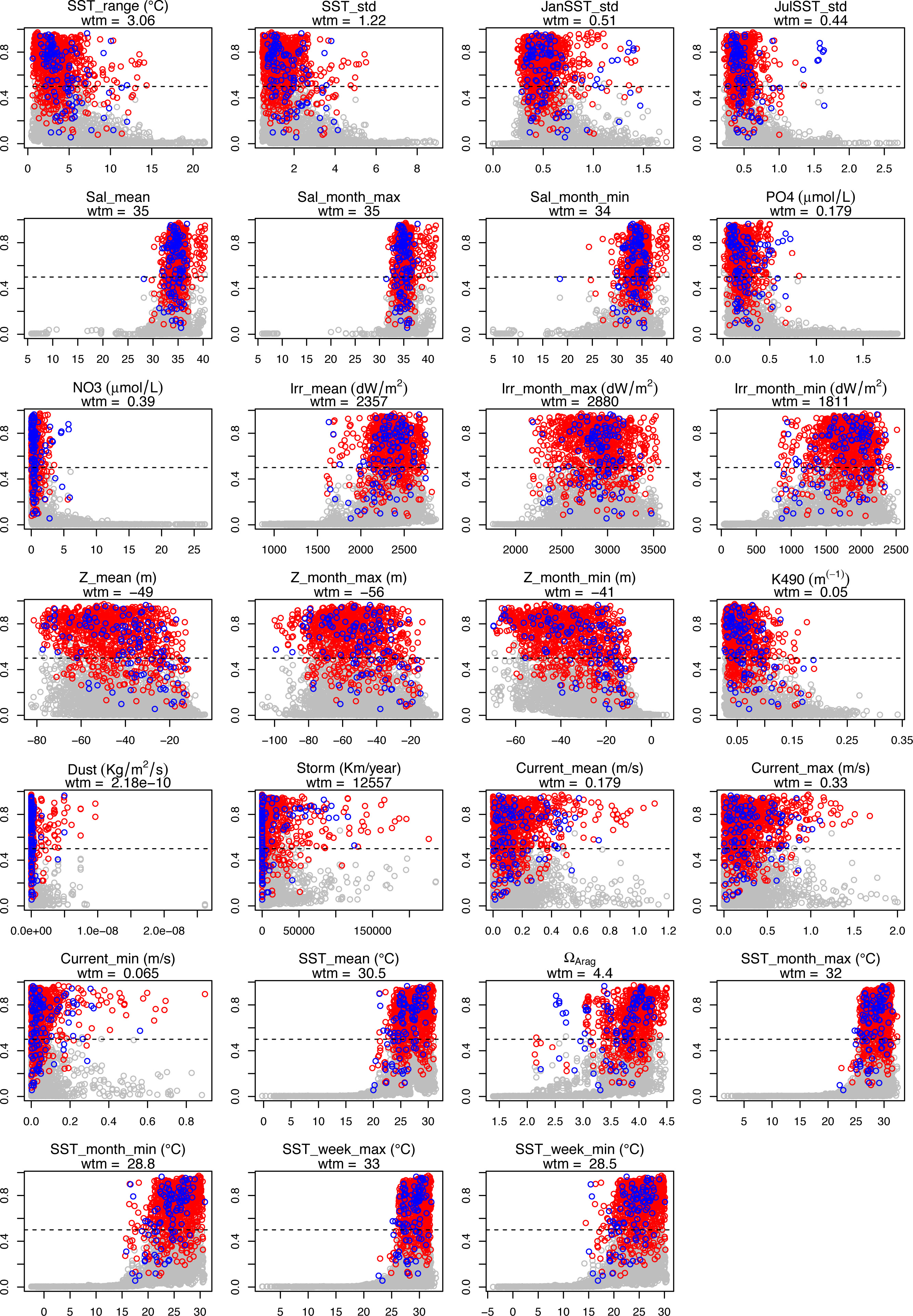
**

**Figure S1.3:** Fitted values (i.e. the y-axis is the model prediction for the cells) with respect to each of the environmental variables for the *BRT_OPT_* model, and colour coded with reef cells (red), non-reef coral communities (blue), and the remaining shallow water grid cells (grey). See Table S1.1 for variable codes. Each variable’s weighted mean is also shown (wtm).

**
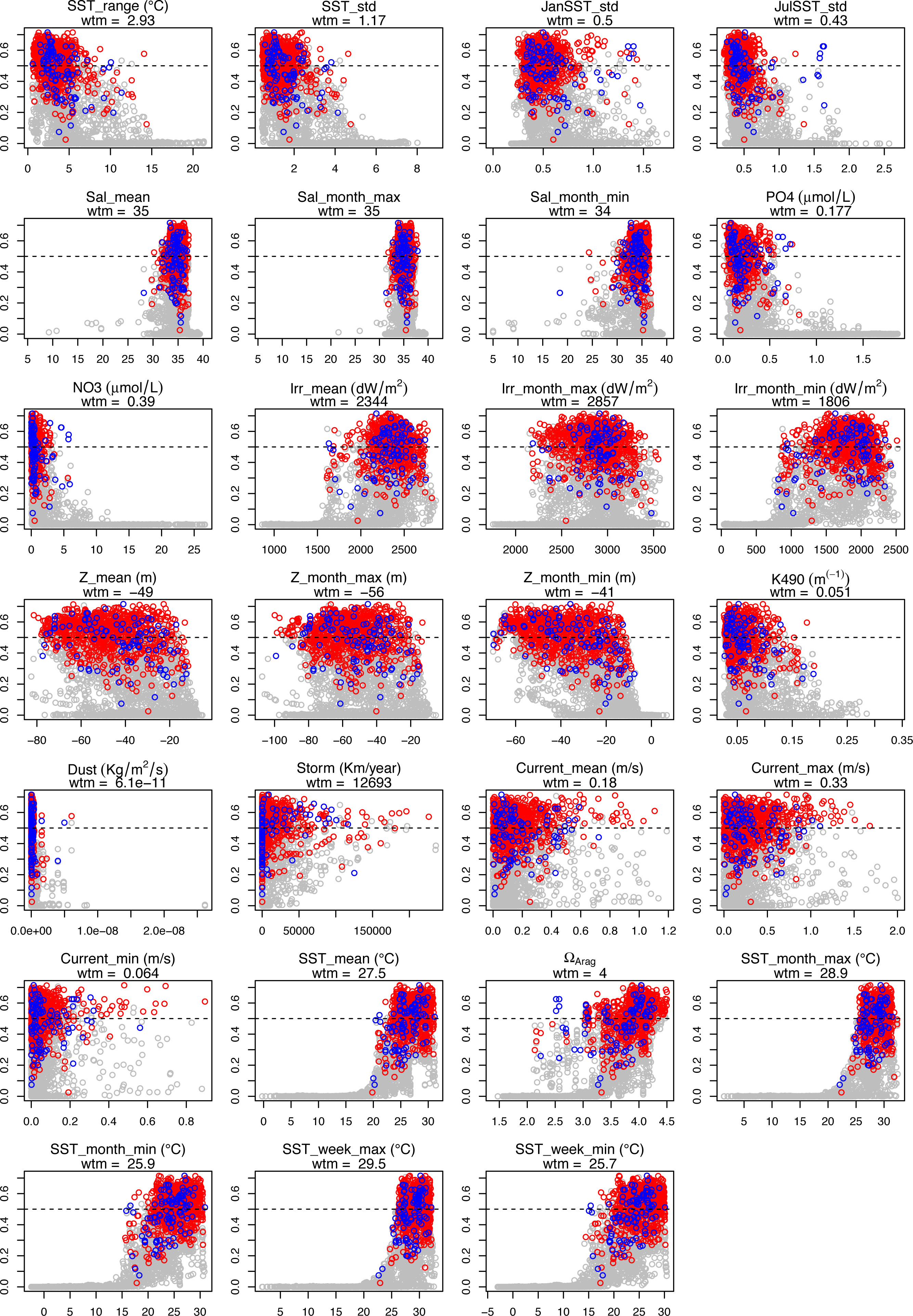
**

**Figure S1.4:** Fitted values plots for the *MaxEnt_OPT_* model, showing reef cells (red), non-reef coral communities (blue), and the remaining shallow water grid cells (grey). Each variable’s weighted mean is also shown (wtm).

**
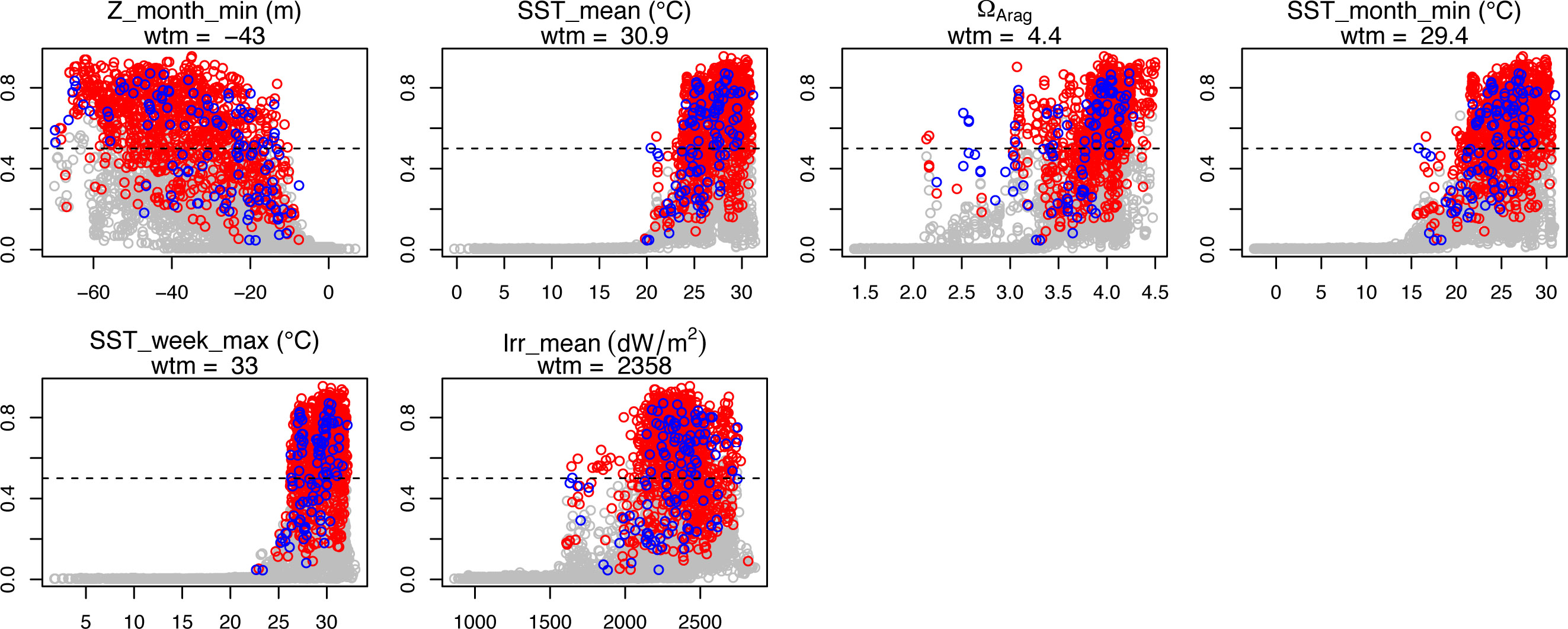
**

**Figure S1.5:** Fitted values for the *BRT_SIM_* model, showing reef cells (red), non-reef coral communities (blue), and the remaining shallow water grid cells (grey).

**
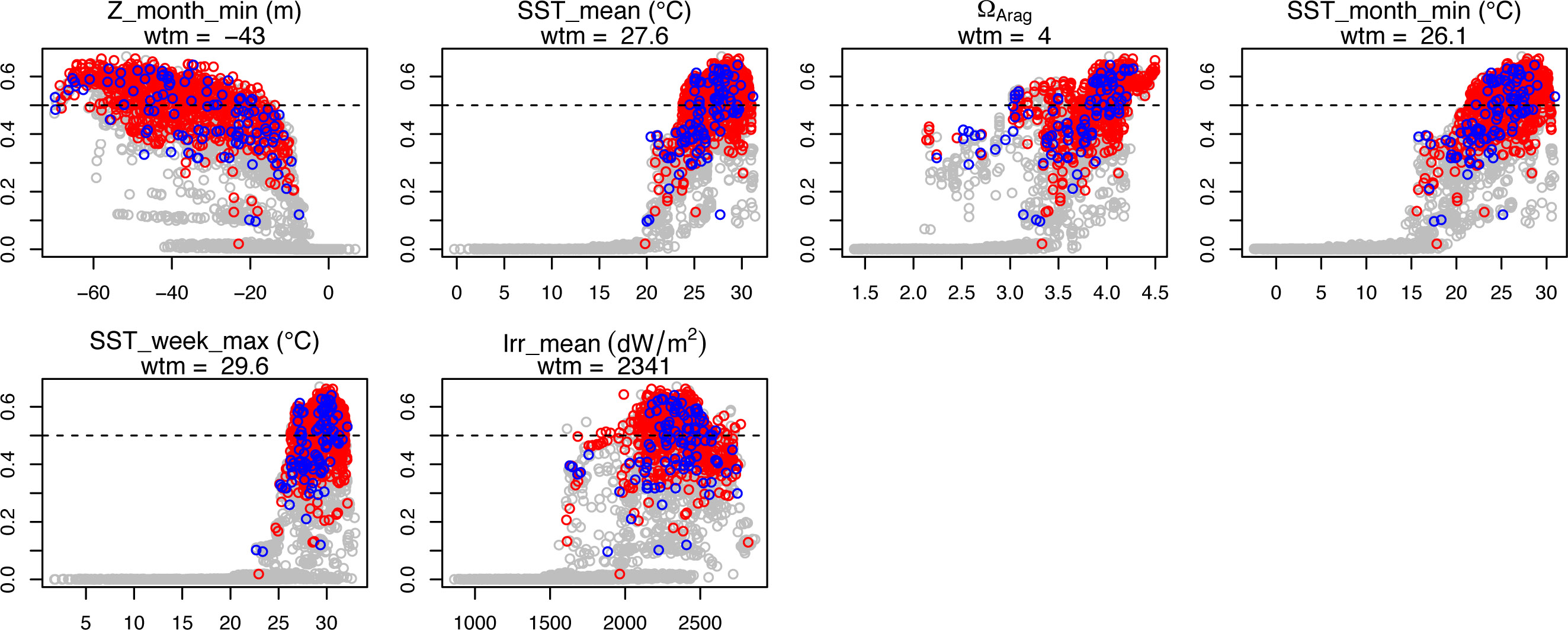
**

**Figure S1.6:** Fitted values plots for the *MaxEnt_SIM_* model, showing reef cells (red), non-reef coral communities (blue), and the remaining shallow water grid cells (grey).

**Figure S1.7:** Marginal response curves for the *BRT_SIM_* model, showing the effect on the model output of variations of a single variable, while keeping the others fixed at their average values. For highly correlated variables this may lead to unrealistic conditions and it may make the curves harder to interpret. The y-axis represents the function assigned in the model, while the x-axis shows the spread of values for each predictive variable.

**Figure S1.8:** Marginal response curves for the *MaxEnt_SIM_* model. MaxEnt’s curves tend to be smoother than those of BRT models, reflecting the higher tendency of Boosted Regression Trees to overfit to training variables.

**Data S2: Extrapolating to novel climates**

When studying habitat suitability for a species or ecosystem under climate change we often run into the problem of having to predict outside the conditions that were used to train the model. It is important in this case to understand how the model is dealing with the out-of-range variables, in order to establish what degree confidence may be put on its projections.

In Figure S2.1 we show the projected changes in the mean annual values of SST and Ω_Arag_ under the A2 scenario, compared with those used to train the model.

**Figure S2.1** (left)**:** Comparison of the values for mean annual SST and Ω_Arag_ used for model training (black) and those projected under the A2 scenario by 2010 (green), 2040 (yellow) and 2070 (red), for all cells within the shallow water mask. As seen there are no SST training values above 31.5 °C.

**Figure S2.2** (below)**:** Cells with values for mean annual SST above 31.5 °C by 2010 (green), 2040 (yellow) and 2070 (red), under the A2 scenario.


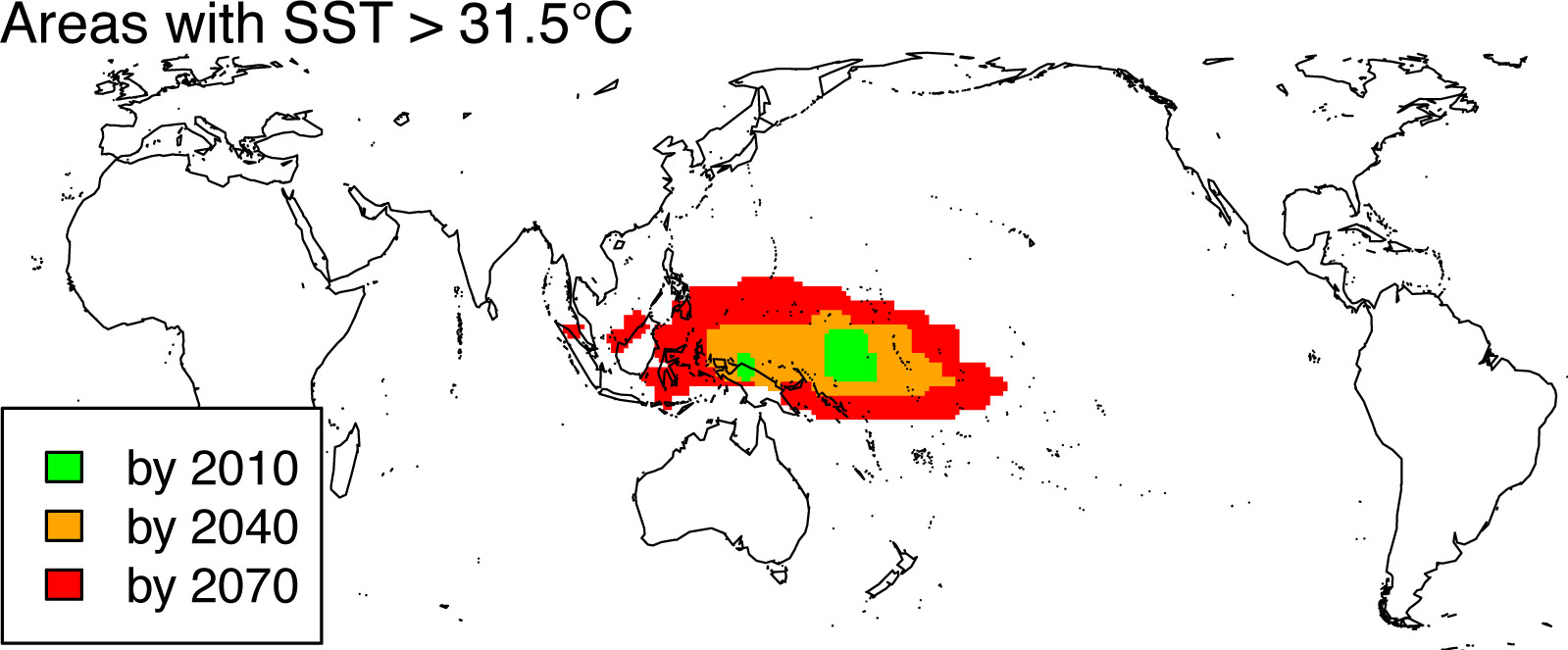


All the BRT and MaxEnt models used in this study deal with the problem of out-of-range extrapolation with a method known in MaxEnt terminology as “clamping” ([Phillips *et al.*, 2006](#_ENREF_8)), for which response is kept constant outside training range (i.e., the models treat variables outside training range as if they were at the limit of the training range). This is the default way of extrapolation in MaxEnt and the only available one for Boosted Trees (e.g., [Elith & Graham, 2009](#_ENREF_2), [Webber *et al.*, 2011](#_ENREF_12)).

MaxEnt provides a number of tests to explore the contribution of clamping to model prediction, which in our case can also be considered to apply to BRT predictions as both algorithms employ the same extrapolation methods and (in the case of the OPTIMAL models and *MaxEnt_SIM_*) obtain similar results. Multivariate Similarity Surfaces ([MESS, described in Elith *et al.*, 2010](#_ENREF_3)) describe how alike conditions for projection are compared with those used in model training. Positive values identify climates similar to those in training, with a score of 100 for average climate conditions in training, while negative values indicate how far out of range the climate of a cell lies, expressed as a the minimum among the predictive variables of how far out the variable is (presented as a fraction of the range at which that variable was found for model training). Figure S2.3a shows the MESS map for 2070 in the A2 scenario, though only comparing the values of the SST and Ω_Arag_ variables (the only ones that have been allowed to change for the predictions of future suitable habitats). Areas outside training range are found in and around the WPWP region, due to the effect of high temperature as mentioned above, and at very high latitudes due to low levels or aragonite saturation (Fig. S2.3b).


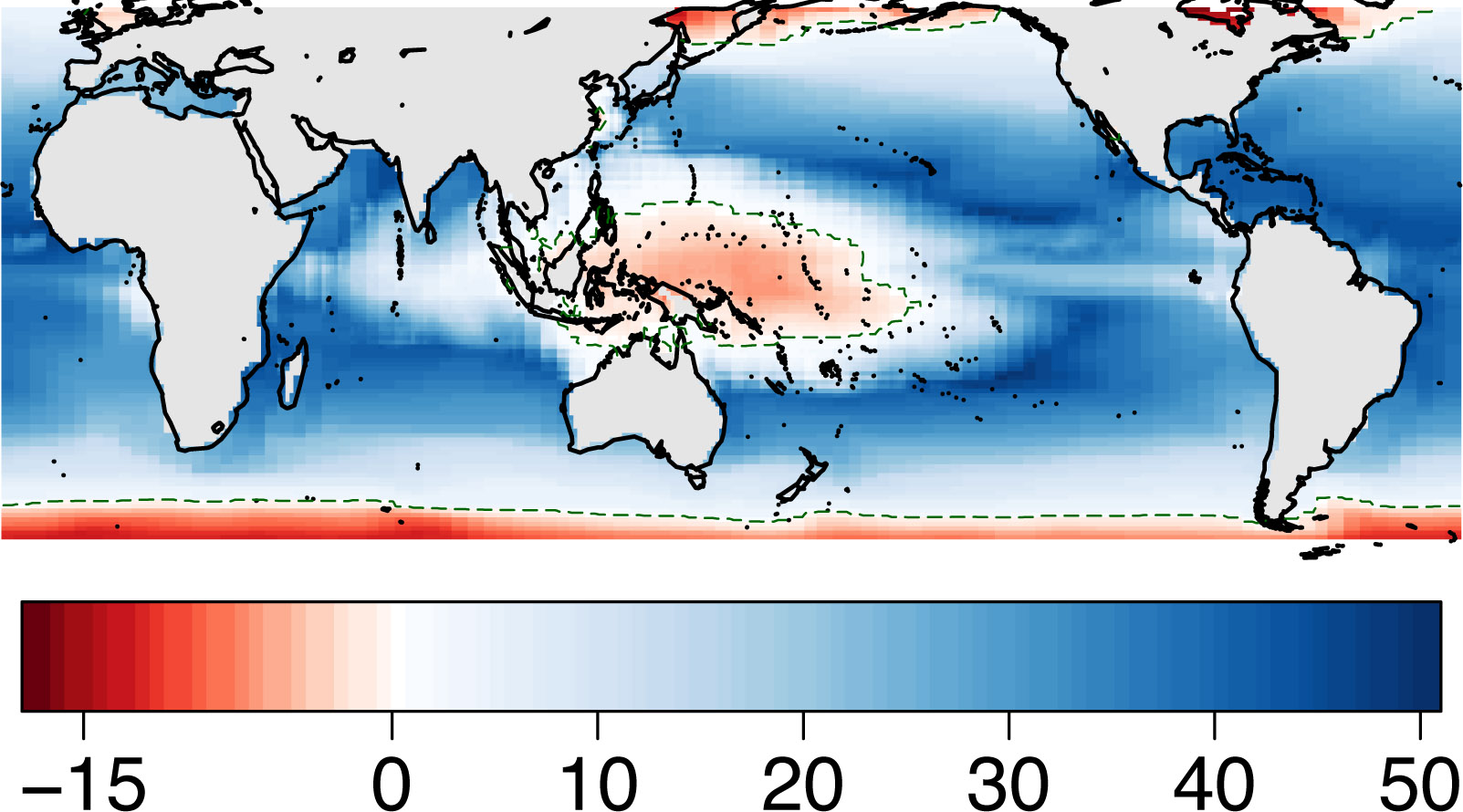
a)

b)

**Figure S2.3:** Multivariate similarity surface (a) comparing the ranges of the SST and Ω_Arag_ variables used in model training with those for the UVic projections for 2070 under the A2 scenario. Negative values (in red) indicate novel climate, while positive scores (blue) indicate similar conditions. The dashed green line marks the 0 value. 3b) shows the single variable which is farthest from training range in all the novel climate areas (those with a negative score in 3a).

Additionally MaxEnt’s projections are provided together with a “clamping map” showing how much effect clamping is having on model predictions (Fig. S2.4), expressed as the difference in the suitability predictions obtained with clamping and those obtained with simple linear extrapolation of the response curves. We see that low aragonite values found at the high latitude limits have no impact on predictions, as those areas already have negligible suitabilities, but we do find a very strong clamping effect in the WPWP region of high SSTs, the same area where both BRT and MaxEnt models are showing a marked decrease in suitabilities (Fig. S2.6, Fig. 5 in the main text). The fact that the

**
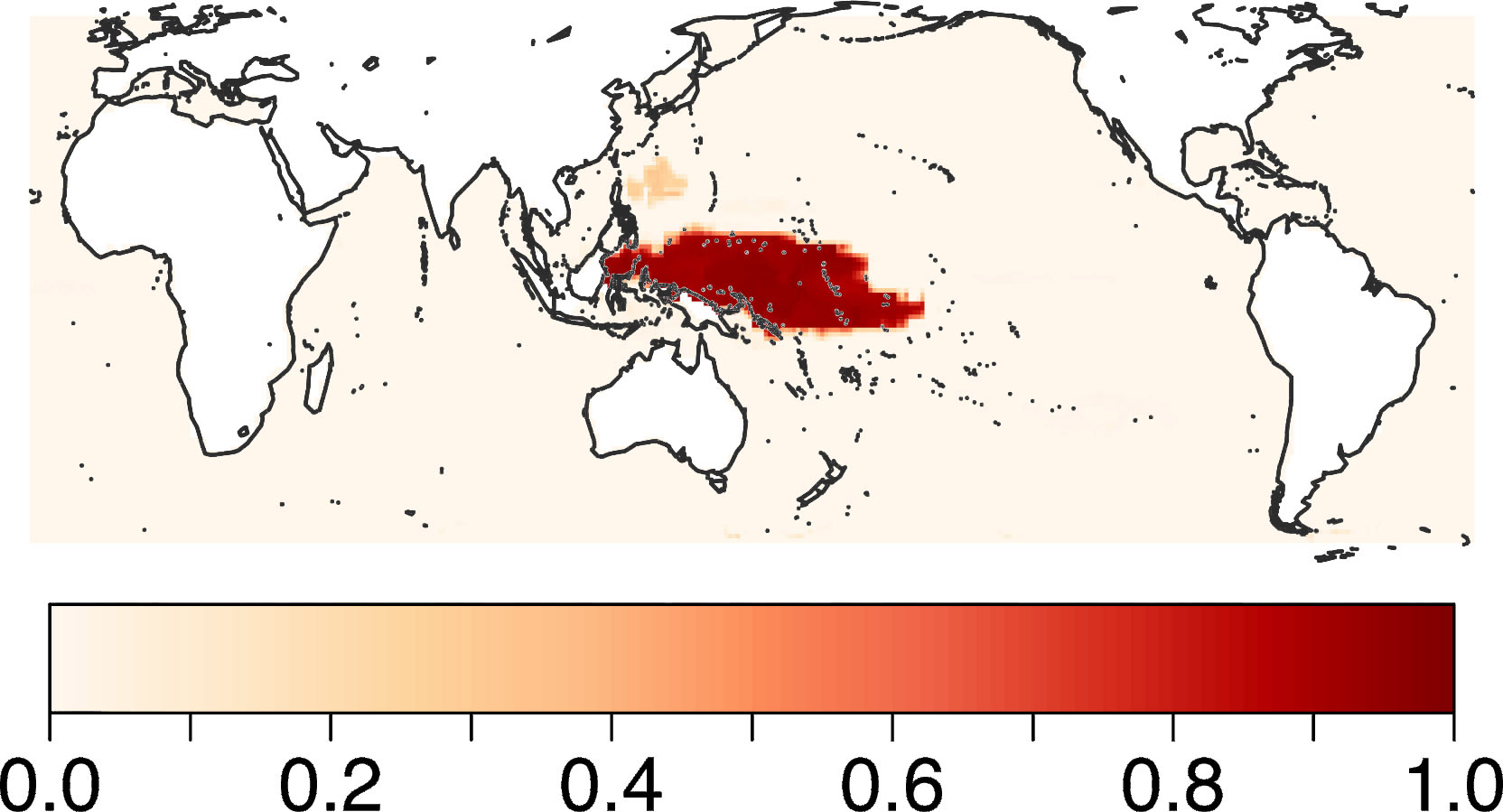
**

**Figure S2.4:** Clamping map of *MaxEnt*_OPT_’s predictions for 2070, A2 scenario. Values close to 1 show areas where the method of extrapolation to novel conditions (in this case, ‘clamping’) is potentially having a strong impact on the predictions. Typically this is taken to mean that predictions are less reliable there.

models project a marked decline there is significant because the dangers of clamping usually lay in overestimating suitability, by maintaining artificially high values in the response curve regardless on how far the conditions get from those found in training. Thus one way commonly used to limit this effect is to subtract the clamping value from the predictions, an option known as “fade-by-clamping” in MaxEnt (used for example in [McKay *et al.*, 2010](#_ENREF_6), [Rödder *et al.*, 2009](#_ENREF_9)). The fact that our future predictions for high SST values are pessimistic even when using the “clamping” option suggests that the limiting effect of high SSTs is already present in training data.

Fig. S2.5 shows the response curves of the *MaxEnt*_OPT_ model for the five temperature variables considered: mean annual, max. and min. monthly means, and max. and min. weekly means, when extrapolated via clamping (S2.5a), linear extrapolation (S2.5b) and fade-by-clamping (S2.5c). Care needs to be taken when interpreting response curves of variables that are so highly correlated, but we can see that the model’s pessimistic predictions for high SST values are more evident in the graphs for the weekly SST extremes. Only the minimum monthly SST variable is increasing markedly at the high temperature limit, an effect that at least within training range is compensated by the combined effect of the other variables. However when extrapolating the response curves beyond training range (S2.5b) this variable alone dominates the prediction (with a logarithmic contribution reaching values two order of magnitude above those of all the other SST variables). This extrapolation is clearly nonsensical and goes against all known physiological responses of coral reef ecosystems (see prediction maps in Fig. S2.6).

**Figure S2.5:** Response curves for the 5 SST variables considered when projecting for future climate. The y-axis of all plots shows the variable’s logarithmic contribution to the model output. In all plots a dashed vertical line marks the high temperature limits present in training conditions. For values higher than this the response curves are extended, either by assuming constant values (a; “clamping”), by linear extrapolation (b) or “fade-by-clamping” (c).

Our projections for novel conditions, obtained by clamping the response curves, represent a conservative estimate of the negative impact of ocean warming, as we are assuming that the effect of SST values beyond training range is just as bad —and not any worse— than that of the highest temperatures already limiting global coral reef distribution.


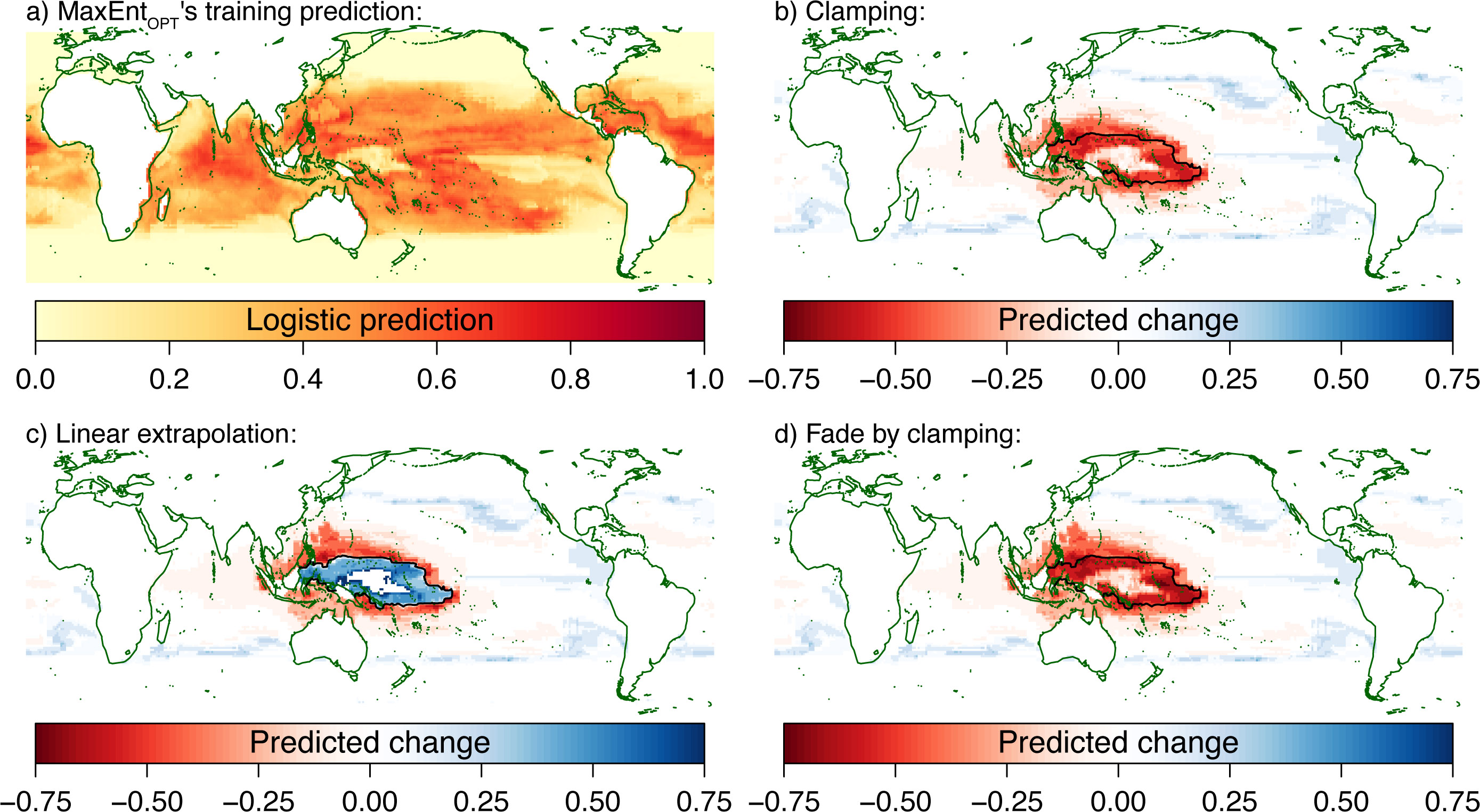


**Figure S2.6** (previous page)**:** *MaxEnt*_OPT_’s training prediction (a) and projected suitability change by 2070 in the A2 scenario when extrapolating via “clamping” (b), linear extrapolation (c) and “fade-by-clamping” (d). In the plots for predicted change levels of clamping of 0.6 or higher are indicated with a black continuous line.

**Data S3: Analysis of model agreement**

Future projections in this work have been obtained with four independent models, developed from two different modeling techniques (BRT and MaxEnt) and two variable sets (OPTIMAL or complete 27-variable set and SIMPLE or reduced 6-variable set). For details see *Methods* and Appendix S1. Due to space limitations it has been necessary to select the results presented in the main manuscript. In this appendix we show the alternative versions for Figs. 2-4 in the main manuscript and discuss model agreement on the projected change by 2070 (Fig. 5 of the main manuscript).


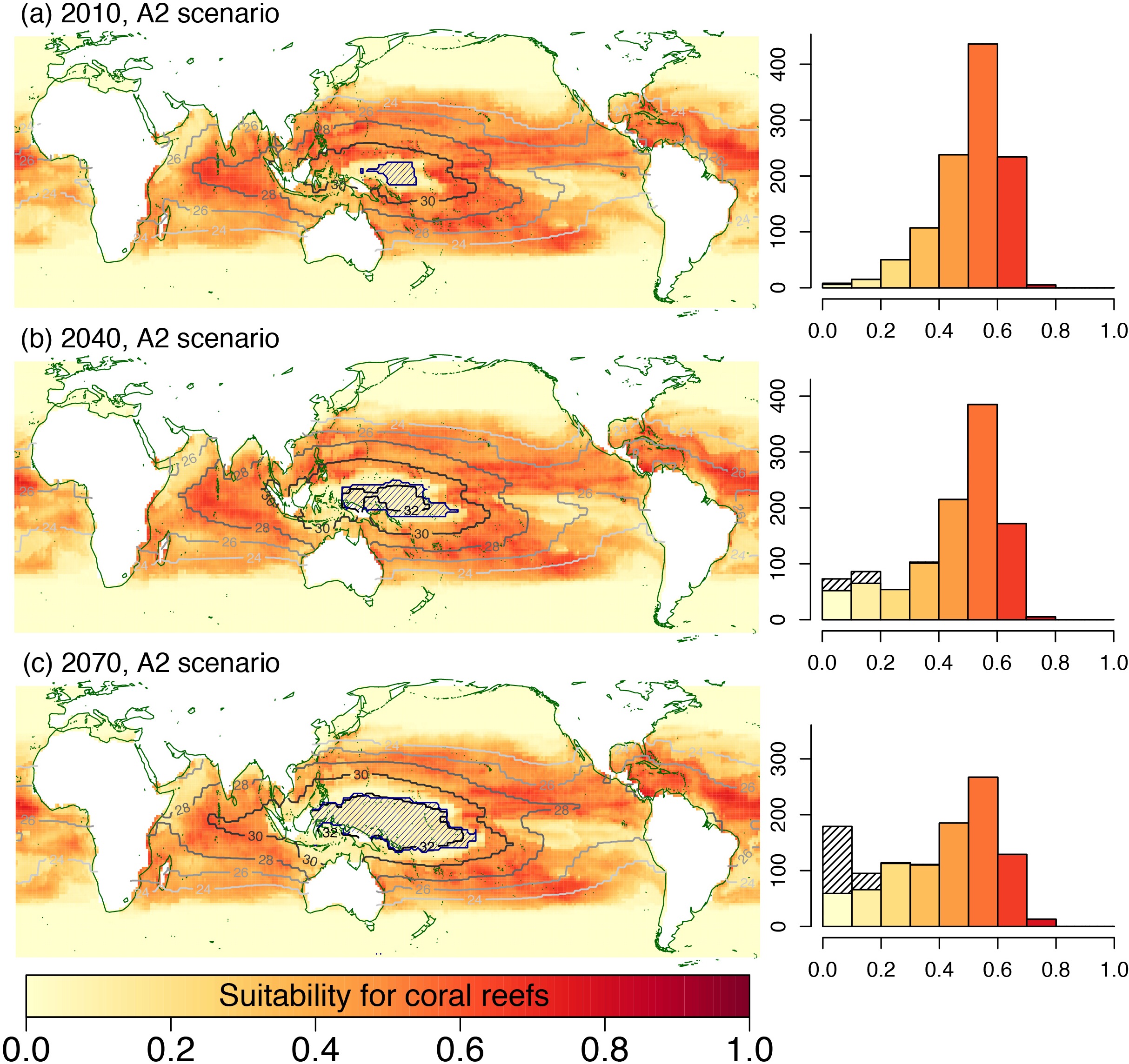


**Figure S3.1:** *MaxEnt_OPT_* model’s predicted changes in suitability for coral reef ecosystems under the A2 scenario when considering future variations in SST variables only, for 2010 (a), 2040 (b) and 2070 (c). The histograms show the predicted suitability values for the 1,124 presence sites of the study grid (i.e. cells currently with reefs according to the ReefBase v2000 data). All environmental fields apart from SST variables were kept constant at their present values (used in model training). This figure complements the *BRT_OPT_* projections shown on the main manuscript (Fig. 2).

Figures S3.1 and S3.2 are the *MaxEnt*_OPT_ versions of Figs. 2 and 3 in the main manuscript (obtained with *BRT*_OPT_), exploring the separate effects of warming acidification respectively. Although MaxEnt distributions for cells currently with coral reefs look different than the results from BRT (since MaxEnt aims to assign an average 0.5 value to presence sites, unlike 1 by BRT), both modeling approaches agree on the relative size of the contributions of warming and acidification, and on the geographical areas most affected.

**
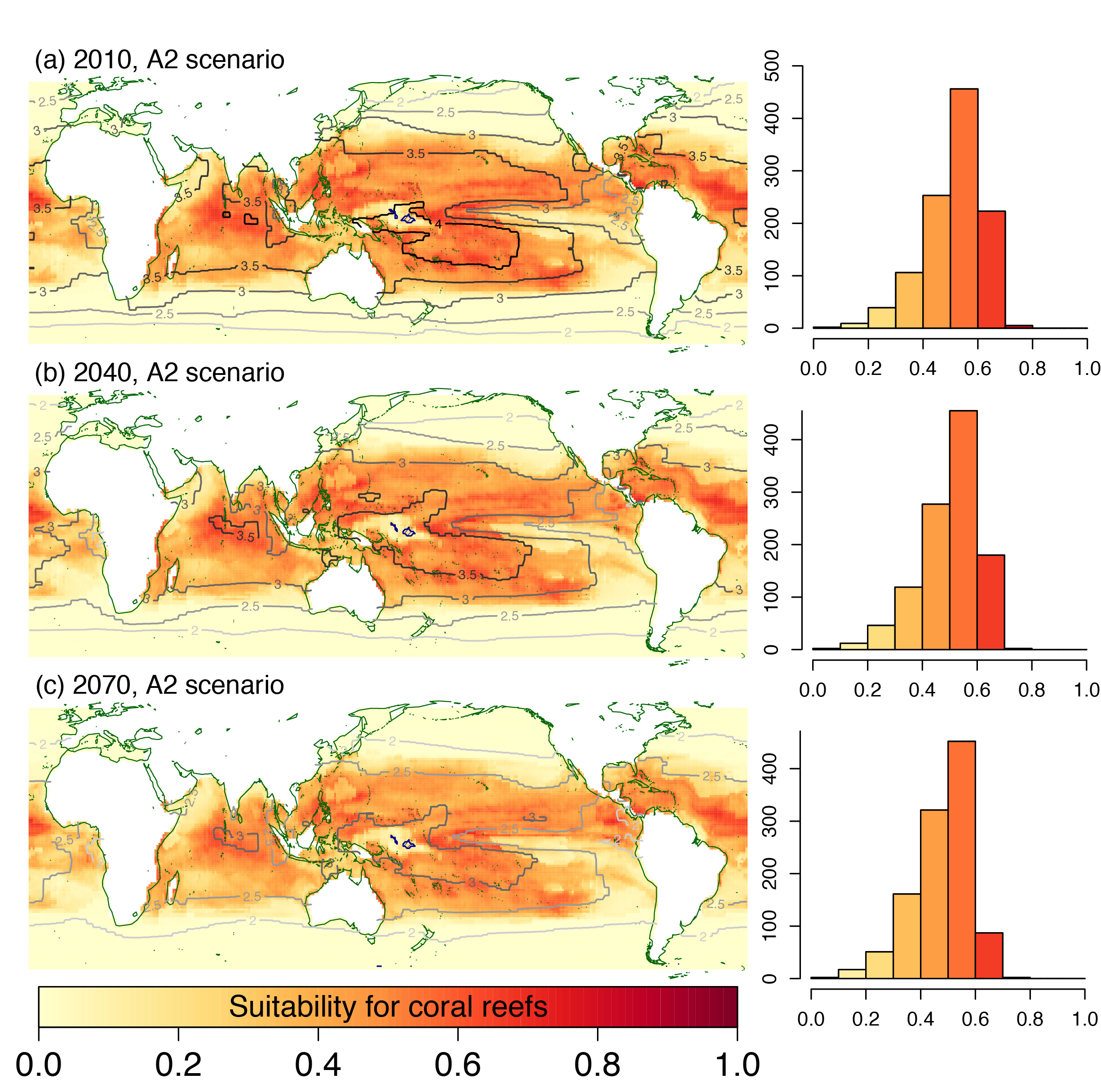
**

**Figure S3.2:** *MaxEnt_OPT_* model’s predicted changes in suitability for coral reef ecosystems under the A2 scenario when considering future variations in aragonite saturation only, for 2010 (a), 2040 (b) and 2070 (c). The histograms show the predicted suitability values for the 1,124 presence sites of the study grid (i.e. cells currently with reefs according to the ReefBase v2000 data). All environmental fields apart from Ω_Arag_ were kept constant at their present values (used in model training). This figure complements the *BRT_OPT_* projections shown on the main manuscript (Fig. 3).

Similarly Fig. S3.3 shows the expected changes in habitat suitability as a function of latitude obtained with *BRT_OPT_* (to be compared to Fig. 6 in the main text, showing *MaxEnt_OPT_*’s results).

**Figure S3.3:** Predicted suitability of coral reef presence as a function of latitude, when changing SST variables only (a), aragonite saturation only (b) and both simultaneously, for the entire ocean (c) and restricted to shallow waters (d). Different colors show variations over time under the A2 scenario. All figures have been made averaging the modelled suitability values in 5° latitudinal bands. This figure corresponds to *BRT_OPT_*’s projections and complements the *MaxEnt_OPT_* results shown on the main manuscript (Fig. 4).

All three previous cases illustrate that although the behavior of the BRT and MaxEnt OPTIMAL models differ, the relative magnitude of the changes (e.g. comparison between the effects of warming and acidification) and main areas of impact (e.g., the WPWP region, under changes in SST variables) are the same. Similarly the main conclusions of the manuscript are robust.

Figure 5 on the main manuscript corresponds to the change in coral reef suitability between 2010 and 2070 under the A2 scenario, averaging the individual predicted changes of 3 of the 4 models, with the exception of *BRT_SIM_*. Looking at the individual model predictions for 2070 (Fig. S3.4), and the numerical comparison of the models’ results (Fig. S3.5), we notice a high level of agreement between the OPTIMAL models (with a correlation of 0.83; see Fig. S3.5a) and the SIMPLE MaxEnt model (correlation of 0.91 with *MaxEnt_OPT_*). However the projected changes obtained by *BRT_SIM_* markedly differ from the rest (e.g. Fig. S3.4d, S3.5b-c). SIMPLE models rely on a reduced number of variables, making their projections more sensitive to changes in those variables than the OPTIMAL versions. This can be clearly seen looking at the results for *MaxEnt_SIM_*, highly correlated to those of *MaxEnt_OPT_* but predicting more extreme responses for either improving or worsening conditions (notice changes in the slope of the correlation curve in Fig. S3.5d for high and low values). They therefore tend to amplify the response to changes. We find that the disparity in the results obtained by *BRT_SIM_*, in particular the appearance of a sequence of red and blue color bands centered on the WPWP area in Fig. S3.4d, reflecting a sequence of high and low suitability projections as temperature increases, are due to the amplification of the effects of over-fitting to SST variables. BRT models, regardless of use of cross-validation in model development, tend to over-fit to training data (e.g., [Couce *et al.*, 2012](#_ENREF_1), [Elith *et al.*, 2008](#_ENREF_4)). While *BRT_SIM_*’s performance on evaluation data is not significantly worse than that of the other models, and may in fact be better than that of *MaxEnt_SIM_* (according to their average ROC curves; Fig. S1.2), its response to novel conditions illustrates the need to validate climate envelope models according to their intended use. We have opted to exclude *BRT_SIM_*’s results from the average computation of Fig. 5 in the main manuscript.


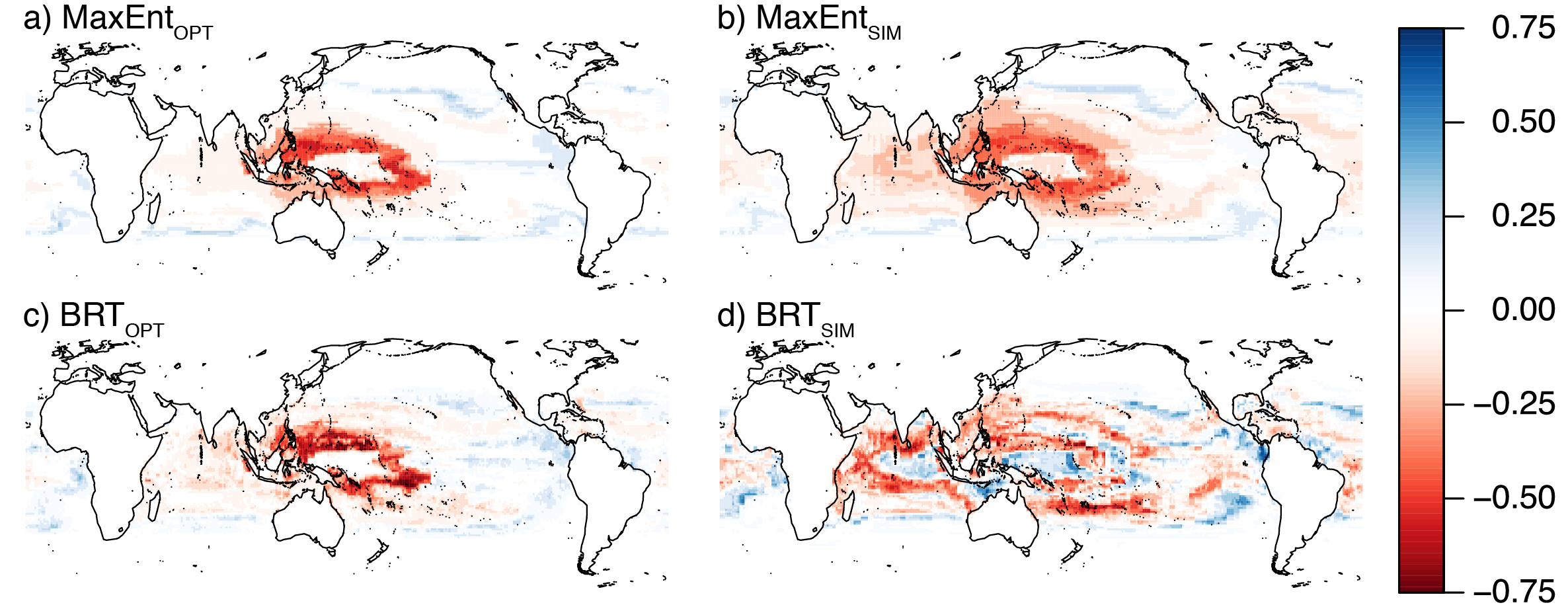
 **Figure S3.4:** Predicted change in suitability for coral reef ecosystems between 2010 and 2070 under the A2 scenario for each of the four individual models: *MaxEnt_OPT_* (a), *MaxEnt_SIM_* (b), *BRT_OPT_* (c) and *BRT_SIM_* (c). Fig. 5 in the main manuscript reflects the average change, excluding the *BRT_SIM_* projections.

**Figure S3.5:** Cell-by-cell comparison of the changes in suitability predicted within the shallow water mask by: *MaxEnt_OPT_* vs *BRT_OPT_* (a); *MaxEnt_SIM_* vs *BRT_SIM_* (b); *BRT_OPT_* vs *BRT_SIM_* (c); and *MaxEnt_OPT_* vs *MaxEnt_SIM_* (d). The Pearson’s correlation coefficient between each pair of variables (‘cor’) in indicated in the bottom right corner of each plot.

The overall similarity between map pairs in Fig. S3.4 was also tested using the Map Comparison Kit software ([version 3.2; Visser & de Nijs, 2006](#_ENREF_11)), by fuzzy numerical comparison of the predicted changes in suitability (Fig. S3.6). Fuzzy numerical comparison is based on certain level of pattern recognition, by comparing not only the values assigned to each individual cell but also –to a lesser extent– those of neighboring cells, and defines the similarity (*s*) between two values *a* and *b* as:

$$s\left( a,b \right)=1- \frac{\left| a-b \right|}{\max(\left| a \right|,\left| b \right|)}$$

In order to aid interpretation, in Fig. S3.6 we have omitted areas were neither of the models being compared predicted a significant change in suitability. We find the level of agreement between *MaxEnt_OPT_* and *BRT_OPT_* (S3.6a) and *MaxEnt_OPT_* and *MaxEnt_SIM_* (S3.6d) is particularly high in areas of projected negative change (e.g., in the central indo-pacific). Agreement in areas of predicted increase suitability (high latitudes and upwelling regions) is not so good. Although all models individually predict a slight improvement in these areas (as seen in Fig. S3.4), the particular patterns for the range expansion show a certain level of divergence, and should thus be regarded less reliable. As an additional test of geographical significance of our projections we have analyzed the areas in which either all 3 or 2 of the models agree at least on the sign of the change (Fig. S3.7).

**
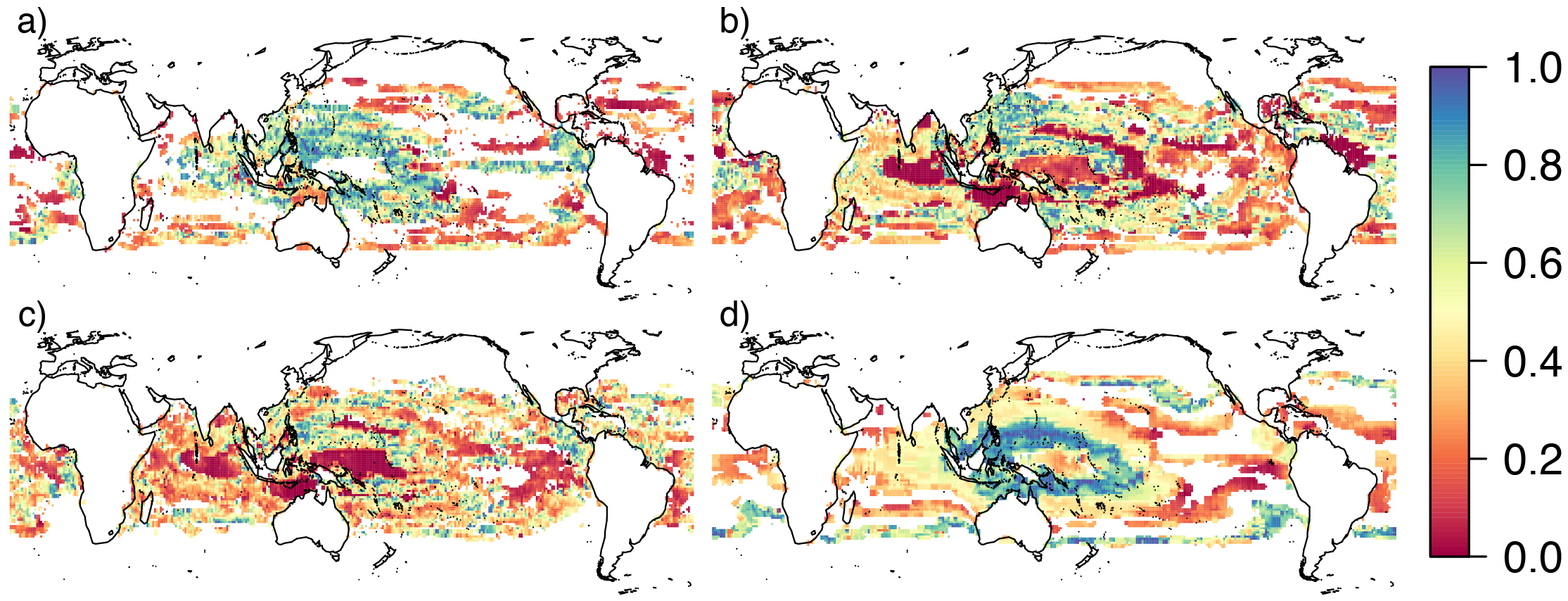
 Figure S3.6:** Fuzzy numerical comparison of the changes in suitability predicted by: *MaxEnt_OPT_* vs *BRT_OPT_* (a); *MaxEnt_SIM_* vs *BRT_SIM_* (b); *BRT_OPT_* vs *BRT_SIM_* (c); and *MaxEnt_OPT_* vs *MaxEnt_SIM_* (d). Green-blue colors indicate good model agreement. Only shown areas where at least one of the models being compared is predicting an absolute change of suitability of at least 0.1. (For the comparison we used a neighbourhood radius of 20 cells, and assumed an exponential decay with a halving distance of 2).


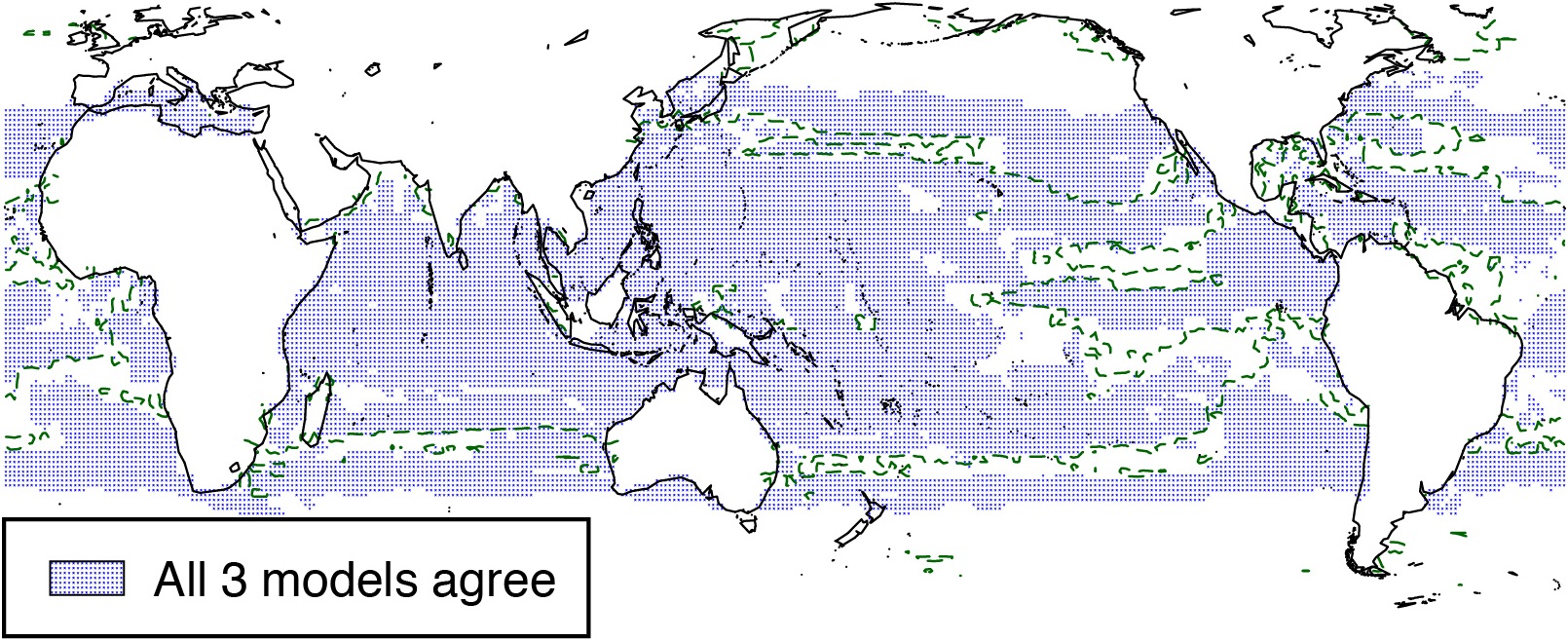


**Figure S3.7:** Significance map for the results shown in Fig. 5 with the average projected change in suitability for coral reef ecosystems between 2010 and 2070. The map shows the areas where all three of the models whose predictions are being averaged agree on the sign of the change in suitability­­­.

**References in the Supporting Information:**

Couce E, Ridgwell A, Hendy EJ (2012) Environmental controls on the global distribution of shallow-water coral reefs. Journal of Biogeography, **39**, 1508-1523.

Elith J, Graham CH (2009) Do they? How do they? WHY do they differ? On finding reasons for differing performances of species distribution models. Ecography, **32**, 66-77.

Elith J, Kearney M, Phillips S (2010) The art of modelling range-shifting species. Methods in Ecology and Evolution, **1**, 330-342.

Elith J, Leathwick JR, Hastie T (2008) A working guide to boosted regression trees. Journal of Animal Ecology, **77**, 802-813.

Friedman JH, Meulman JJ (2003) Multiple additive regression trees with application in epidemiology. Statistics in Medicine, **22**, 1365-1381.

Mckay B, Mays H, Peng Y-W, Kozak K, Yao C-T, Yuan H-W (2010) Recent range-wide demographic expansion in a Taiwan endemic montane bird, Steere's Liocichla (*Liocichla steerii*). BMC Evolutionary Biology, **10**, 71.

Phillips S (2006) A brief tutorial on Maxent. AT&T Research. Available at: <http://www.cs.princeton.edu/~schapire/maxent/tutorial/tutorial.doc>.

Phillips SJ, Anderson RP, Schapire RE (2006) Maximum entropy modeling of species geographic distributions. Ecological Modelling, **190**, 231-259.

Rödder D, Kielgast J, Bielby J *et al.* (2009) Global Amphibian Extinction Risk Assessment for the Panzootic Chytrid Fungus. Diversity, **1**, 52-66.

Swets JA (1988) Measuring the Accuracy of Diagnostic Systems. Science, **240**, 1285-1293.

Visser H, De Nijs T (2006) The Map Comparison Kit. Environmental Modelling & Software, **21**, 346-358.

Webber BL, Yates CJ, Le Maitre DC *et al.* (2011) Modelling horses for novel climate courses: insights from projecting potential distributions of native and alien Australian acacias with correlative and mechanistic models. Diversity and Distributions, **17**, 978-1000.
